# Immunotherapies targeting stimulatory pathways and beyond

**DOI:** 10.1186/s13045-021-01085-3

**Published:** 2021-05-12

**Authors:** Julian A. Marin-Acevedo, ErinMarie O. Kimbrough, Rami Manochakian, Yujie Zhao, Yanyan Lou

**Affiliations:** 1 Department of Hematology and Oncology, H. Lee Moffitt Cancer Center, 12902 USF Magnolia Drive, Tampa, 33612 FL USA; 2 Division of Hematology and Oncology, Mayo Clinic, 4500 San Pablo Road S., Jacksonville, FL 32224 USA

**Keywords:** Cancer, Immunotherapy, Tumor microenvironment, Immune evasion, Cytotoxic T lymphocytes, Immune checkpoint, Co-stimulatory pathway

## Abstract

Co-stimulatory and co-inhibitory molecules play a critical role in T cell function. Tumor cells escape immune surveillance by promoting immunosuppression. Immunotherapy targeting inhibitory molecules like anti-CTLA-4 and anti-PD-1/PD-L1 were developed to overcome these immunosuppressive effects. These agents have demonstrated remarkable, durable responses in a small subset of patients. The other mechanisms for enhancing anti-tumor activities are to target the stimulatory pathways that are expressed on T cells or other immune cells. In this review, we summarize current phase I/II clinical trials evaluating novel immunotherapies targeting stimulatory pathways and outline their advantages, limitations, and future directions.

## Introduction

Cancer cells create an immunosuppressive milieu known as the tumor microenvironment (TME) to evade immune recognition. They recruit immunomodulatory cells including regulatory T cells (Tregs) and myeloid suppressor cells that allow for tumor growth and alter immune function [1]. Chronic inflammation leads to T cell exhaustion and apoptosis within the TME. Cancer cells downregulate surface antigens to avoid immune recognition and increase the expression and secretion of molecules that enhance immunosuppressive pathways, i.e., cytotoxic T lymphocyte-associated molecule-4 (CTLA-4) and programmed cell death receptor-1 (PD-1)/ligand (PD-L1) [1–3].

**Fig. 1:**
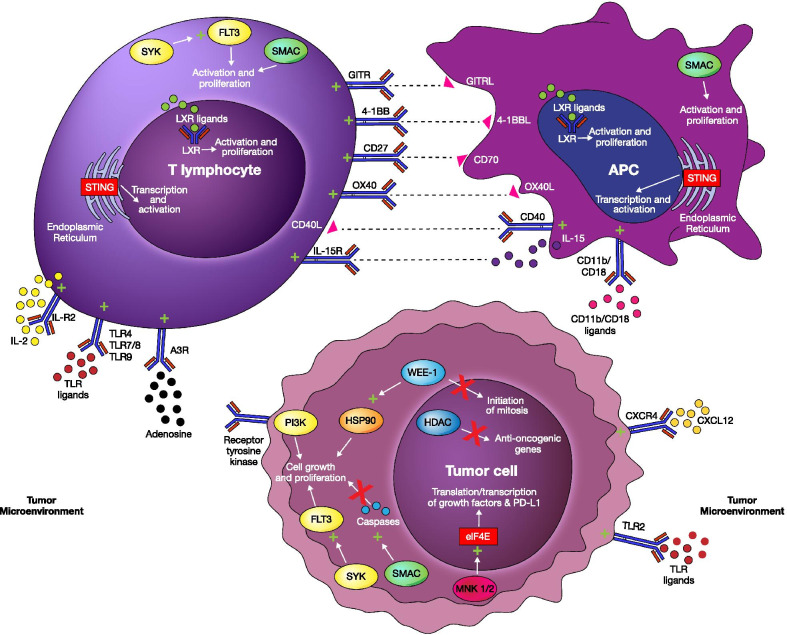
Stimulatory immune checkpoints and other stimulatory targets and their effects on immune-cell function and cancer cells

Immunotherapy enhances the host immune system to fight cancer. Immune checkpoint inhibitors including anti-CTLA-4 and anti-PD-1/PD-L1 agents are well known and heavily utilized forms of immunotherapy [2]. These agents have demonstrated remarkable, durable responses in a small subset of patients [4]. In an attempt to increase the efficacy and broaden the application of existing therapies, novel strategies utilizing immune checkpoints with stimulatory properties are in development [5, 6]. In addition, therapies which activate the immune response and improve tumor recognition within the TME are being investigated [6–8]. In this review, we focus on new investigational agents in phase I and II clinical trials that have emerged over the last 3 years. The therapies outlined target stimulatory immune checkpoint pathways, the TME, or indirectly affect and enhance the function of immune cells. Figure 1 depicts these targets and their mechanism of action. This is an update from a previous review of novel investigational molecules in immune checkpoint therapy published in 2018 [6].

## Methodology

We conducted a PubMed search using the keywords and MeSH terms immunotherapy, immune checkpoint therapy, and immune checkpoint stimulators. In addition, we reviewed the meeting abstracts and posters from the American Society of Clinical Oncology (ASCO), the American Association for Cancer Research (AACR), and information from ClinicalTrials.gov. We included data from February 1, 2018 through June 1, 2020 and focused on phase I and phase II clinical trials developing agents in the field of immune checkpoint therapy. Our data includes preliminary results of ongoing trials, as well as, completed trials. We evaluated drugs that directly enhance stimulatory immune checkpoints. We also included experimental agents that indirectly activate the immune system by altering the TME or by manipulating pathways that lead to immune activation. We excluded advanced clinical trials (e.g., stage III or more), those that explored inhibitory immune checkpoints such as CTLA-4, PD-1 and/or PD-L1, vaccines, viruses, immune cell therapy, and clinical trials involving the pediatric population. We included a total of 35 phase I, 14 phase I/II, and 5 phase II clinical trials in this review. A summary of the trials is included in Table 1.

**Table 1:** Summary of ongoing phase I, I/II, and II clinical trials of novel immunotherapy agents

Category	Target	Drug	Trial	Phase	Type of tumor	Clinical efficacy	Safety	Comments
T cell co-stimulatory targets	CD27	Varlilumab	NCT01460134	I	Advanced solid malignancies	*N* = 25 ORR 4% and DCR 36% with 1 PR and 8 SD	No DLTs reported	Used as monotherapy
			NCT02335918	I/II	Advanced solid malignancies	*N* = 90 ORR 8% and DCR 37% with 7 PR and 26 SD	5 serious AEs: acute kidney injury, mixed motor neuropathy, pneumonitis, small bowel obstruction, and acute hepatitis	Used with nivolumab
	CD70	ARGX-110	NCT01813539	I/II	Advanced, CD70 positive solid or hematologic malignancies	*N* = 26 DCR 54%, 14 SD with a mean duration of 3.7 months	No DLTs reported	Used as monotherapy
	CD40/ CD40L	SEA-CD40	NCT02376699	I	Advanced solid malignancies	*N* = 34 ORR 3% and DCR 32% with 1 PR and 10 SD	5 cases of DLTs, all infusion-related reactions Serious AEs included dyspnea (27%), and headache (27%)	Used as monotherapy
		CP-870,893	NCT01103635	I	Metastatic melanoma	*N* = 22 ORR 27%, DCR 59% with 2 CR, 4 PR, and 7 SD Median PFS was 3.2 months	2 cases DLTs (immune colitis) Serious AEs: one grade 3 CRS	Used with tremelimumab
		JNJ-64457107 (JNJ-107)	NCT02829099	I	Advanced solid malignancies	*N* = 95 ORR 1%, DCR 25% with 1 PR and 23 SD	2 cases of DLTs: 1 grade 3 headache and 1 grade 3 elevation of transaminases with grade 2 hyperbilirubinemia	Used as monotherapy
		APX005M	NCT03123783	I/II	Advanced melanoma and immunotherapy-naïve NSCLC	Phase I: *N* = 9 ORR 22%, DCR 67% with 2 PR, 4 SD, 3 PD Phase II: *N* = 10 ORR 20%, DCR 40% with 2 PR, 2 SD, 6 PD	No DLTs reported	Used with nivolumab
			NCT02706353	I/II	Metastatic melanoma	–	–	Ongoing
	4-1BB/ 4-1BBL	Utomilumab (PF05082566)	NCT02179918	I	Advanced solid malignancies	*N* = 23 ORR 26%, DCR 70% with 2 CR, 4 PR, 10 SD	No DLTs reported	Used with pembrolizumab
		ADG106	NCT03802955	I	Advanced solid malignancies or relapsed and refractory NHL	*N* = 14 ORR 0%, DCR 57% with 8 SD	No DLTs reported	Used as monotherapy
	OX40/ OX40L	MEDI0562	NCT02705482	I	Advanced solid malignancies	Durvalumab cohort: *N* = 26 ORR 12%, DCR 46% with 3 PR, 9 SD Tremelimumab cohort: *N* = 31 ORR 0%, DCR 29% with 9 SD	5 cases of DLTs (2 with durvalumab and 3 with tremelimumab), no specifics provided 2 grade 5 AEs related to MEDI0562: renal failure (durvalumab cohort) and multiorgan failure (tremelimumab cohort)	Used with durvalumab or tremelimumab
		GSK3174998 (GSK998)	NCT02528357	I	Advanced solid malignancies	*N* = 138 ORR 7%, DCR 14% with 2 CR, 8 PR, 10 SD	2 DLTs in the combination group: 1 grade 3 pleural effusion and 1 grade 1 myocarditis	Used as monotherapy or with pembrolizumab
		ATOR-1015	NCT03782467	I	Advanced solid malignancies	No specifics provided. 9/14 patients have discontinued therapy: clinical deterioration (*n* = 6), death from disease (*n* = 2), or PD (*n* = 1)	No DLTs reported	Used as monotherapy
		mRNA-2416	NCT03323398	I	Advanced solid or hematologic malignancies	Monotherapy: *N* = 39 ORR 0%, DCR of 15% with 6 SD lasting ≥ 14 weeks	No DLTs reported	Used as monotherapy or with durvalumab
	GITR/ GITRL	MK-1248	NCT02553499	I	Advanced solid malignancies	Monotherapy: *N* = 20 ORR 0% and DCR 15% with 3 SD Combination: *N* = 17 ORR 18% and DCR 47% with 1 CR, 2 PR, 3 SD	No DLTs reported	Used as monotherapy or with pembrolizumab
		AMG 228	NCT02437916	I	Advanced solid malignancies	*N* = 27 ORR 0%, DCR 23% with 7 SD and 20 PD	No DLTs reported 1 grade 5 pneumonitis	Used as monotherapy
		BMS-986156	NCT02598960	I/II	Advanced solid malignancies	Monotherapy: *N* = 34 ORR 0%, DCR 32% with 11 SD, 18 PD, 5 non-evaluable disease Combination: *N* = 258 ORR 7%, DCR 41% with 2 CR, 19 PR, 84 SD, 117 PD, 18 non-evaluable disease	1 DLT: Grade 4 CK elevation	Used as monotherapy or with nivolumab
Other Direct Immune Stimulatory Targets	LXR	RGX-104	NCT02922764	I	Advanced solid or hematologic malignancies	Monotherapy: *N* = 12 DCR 42% with 5 confirmed SD, 1 presumptive PR Combination: *N* = 11 ORR 22%, DCR 66% with 2 CR, 4 SD	Neutropenia was a DLT reported only in the combination cohort No additional details provided	Used as monotherapy or with docetaxel
	IL-2/ IL-2R	RO6874281	NCT02627274	I	Advancedsolid malignancies	No specifics are provided. Long-lasting responses in 3/35 patients	No DLTs reported	Used as monotherapy
		hu14.18-IL2	NCT00590824	II	Resectable stage III or IV melanoma	No details are provided on responses for 18 patients (*n* = 11 neoadjuvant, and *n* = 7 adjuvant) 6 patients had a median RFS of 5.7 months, a 24-month RFS rate 39% and a 24-month OS of 65%	6 toxicities leading to dose-adjustments: hypotension (n = 3), syncope (n = 1), elevated transaminases (n = 2), and elevated serum creatinine (n = 1)	Used as monotherapy
		ALKS 4230	NCT02799095	I/II	Advanced solid malignancies	Monotherapy: *N* = 14 ORR 0%, DCR 57% with 8 SD Combination: *N* = 11 ORR 9%, DCR 73% with 1 PR,7 SD	No DLTs reported	Used as monotherapy and with pembrolizumab
		Bempegaldesleukin (NKTR-214 – BEMPEG)	NCT02983045	I/II	Advanced solid malignancies	*N* = 23 ORR 48%, DCR 70% with 4 CR, 7 PR, 5 SD	No DLTs reported 9% stopped therapy from AEs	Used with nivolumab
					Previously untreated stage IV melanoma patients	*N* = 38 ORR 53% with 13 CR and 7 PR Median duration of response was not reached	No DLTs reported 10% stopped therapy from AEs	
		IRX-2	NCT03758781	I	Advanced solid tumors	–	–	Ongoing
	IL-15	ALT-803	NCT02523469	I	Stage III or IV NSCLC	*N* = 21 ORR 29%, DCR 76% with 6 PR, 10 SD Median PFS: 9.4 months Median OS: 17.4 months	No DLTs reported	Used with nivolumab
			NCT01885897	I/II	Relapsed hematologic malignancies after allogenic bone marrow transplant	*N* = 33 ORR 6%, DCR 15%, with 1 CR (lasted 7 months), 1 PR (lasted 5 months), 3 SD (lasted ≥ 2 months)	No DLTs reported	Used as monotherapy
		n-803	NCT03381586	I	Healthy volunteers	–	No DLTs reported	Used as monotherapy
		rhIL-15	NCT03388632	I	Advanced solid malignancies	–	–	Ongoing
	A3R	Namodenoson (CF102)	NCT02128958	II	Advanced, refractory HCC and Child–Pugh class B	*N* = 34 ORR 9% with 3 OR. No specifics about the type of responses are available OS 4.1 months	No grade 4–5 AEs	Used as monotherapy
	CD11b	GB1275	NCT04060342	I/II	Advanced solid malignancies	*N* = 22 DCR 32% with 7 SD (4 monotherapy and 3 combination cohort)	No DLTs reported	Used as monotherapy or with pembrolizumab or nab-paclitaxel and gemcitabine
	STING	MIW815 (ADU-S100)	NCT03172936	I	Advanced solid malignancies and lymphomas	No specifics are provided regarding responses At time of cut-off, 74% of patients (*n* = 49) were unenrolled: PD (*n* = 28), physician decision (*n* = 18), AEs (*n* = 2), or death (*n* = 1)	No DLTs reported	Used in combination with spartalizumab
		SB 11,285	NCT04096638	I	Advanced solid malignancies	–	–	Ongoing
	TLR-2	Tomaralimab (OPN-305)	NCT02363491	I/II	Heavily pretreated patients with low and intermediate risk MDS	*N* = 22 ORR 50%, DCR 73% with 6 CR, 5 PR, 5 SD	No DLTs No additional toxicity data was provided	Used as monotherapy
	TLR-4	G100	NCT02180698	I	Unresectable or metastatic soft tissue sarcomas	*N* = 14 ORR 14%, DCR 100% with 1 CR, 1 PR, 11 SD	There were no grade 3–5 AEs reported No additional safety data was provided	Intratumoral monotherapy
	TLR-7	NJH395	NCT03696771	I	Non-breast HER2 + advanced malignancies	*N* = 18 ORR 0%, DCR 50% with no CR or PR, 9 SD	5 DLTs: 3 increased liver enzymes, 1 aseptic meningitis, and 1 meningism	Used as monotherapy
	TLR-8	Motolimod (VTX-2337)	NCT02124850	I	Previously untreated stage II, III, and IV HNSCC	No clinical efficacy reported	No DLTs reported	Used with cetuximab
			NCT01836029	II	Recurrent/ metastatic HNSCC	Motolimod: *N* = 100 ORR 40%, DCR 62% with 2 CR, 36 PR, 22 SD. Median PFS 6.1 months and median OS 13.5 months Placebo: *N* = 95 ORR 34%, DCR 58% with 5 CR, 27 PR, and 23 SD. PFS 5.9 months, OS 11.3	Serious AEs: vomiting (6%), pneumonia (6%), and dehydration (6%)	Combined with platinum therapy, fluorouracil, and cetuximab
	TLR-7 and 8	MEDI9197	NCT02556463	I	Advanced solid malignancies	MEDI9197 monotherapy: *N* =35 ORR 0%, DCR 29% with 0 CR, 0 PR, and 10 SD MEDI9197 with durvalumab: *N* = 17 ORR 0%, DCR 18% with 0 CR, 0 PR, 3 SD	Monotherapy: 2 DLTs from CRS Combination: 1 DLT from hemorrhagic shock from a ruptured liver metastasis	Intratumoral use with or without durvalumab and/or radiation therapy
		NKTR-262	NCT03435640	I	Advanced solid malignancies	*N* = 17 ORR not provided, DCR 41%. Unknown specific number of CR, PR, SD At least 2 PRs mentioned	1 DLT: transaminitis	Used with bempegaldesleukin
	TLR-9	Tilsotolimod (IMO-2125)	NCT03052205	I	Advanced solid malignancies	*N* = 51 ORR 0%, DCR 29% with 15 SD	No DLTs reported	Used as intratumoral monotherapy
			NCT02644967	I/II				
		Lefitolimod (MGN1703)	NCT02200081	II	Extensive-stage SCLC	*N* = 59 ORR 12%, DCR 51%, 0 CR, 7 PR2, SD 23	No grade 5 AEs 5 with grade 4 neutropenia	Maintenance monotherapy
			NCT02668770	I	Advanced solid malignancies	–	–	Ongoing
		Cavrotolimod (AST-008)	NCT03684785	I/II	Advanced solid malignancies	–	–	Ongoing
	SMAC/ IAP	Birinapant	NCT02587962	I/II	Advanced solid malignancies	*N* = 18 ORR 11% DCR 22% with 2 PR, 2 SD	1 DLT: grade 3 elevation of transaminases	Used with pembrolizumab
Indirect Immune activators	CXCL12	NOX-A12	NCT03168139	I/II	Metastatic pancreatic and MSS CRC	*N* = 20 ORR 0%, DCR 25% with 5 SD Median PFS: 1.87 months OS: 42% at 6 months and 22% at 12 months	No specifics provided but attributed to pembrolizumab	Used as monotherapy and with pembrolizumab
	CXCR4	Balixafortide	NCT01837095	I	Metastatic, HER-2 negative, CXCR4 positive BC	*N* = 56 ORR 30%, median duration of 3.2 months Clinical benefit rate 44%, median duration 6.9 months DCR 76% There were 16 PR, 25 SD (8 of which lasted > 6 months)	No DLTs reported Serious AEs in 38%: febrile neutropenia (9%), urinary tract infection (5%), and pneumonia (4%)	Used with eribulin
	PI3K	IPI-549	NCT02637531	I	Advanced solid malignancies	*N* = 30 ORR 7% with 2 PR at 8 weeks	2 DLTs: one grade 3 rash and one grade 3 transaminitis	Used with nivolumab
		SAR260301	NCT01673737	I	Advanced solid malignancies	*N* = 19 ORR 0%, DCR 26% with 5 SD	2 DLTs: one grade 3 pneumonitis and one grade 3 GGT elevation	Used as monotherapy
	SYK/ FLT3	TAK-659	NCT02834247	I	Advanced solid malignancies	*N* = 19 ORR 5%, DCR 63% with 1 PR and 11 PD	3 DLTs: one grade 3 fever, one myocarditis and one left ventricular dysfunction (both thought to be due to nivolumab)	Used with nivolumab
	MNK 1/2	Tomivosertib (eFT508)	NCT03616834	II	Advanced solid malignancies	*N* = 39 ORR 5%, DCR 46% with 3 PR and 15 SD 7 NSCLC patients had DFP of ≥ 24 weeks	7 DLTs: hypersensitivity, hepatic toxicity, and constipation 4 grade 5 AEs but none were attributed to tomivesertib	Used with other ICIs
	HDAC	Vorinostat	NCT02619253	I	Advanced, refractory urothelial, renal, and prostate carcinoma	*N* = 37 ORR 0%, DCR 5% with 2 PR PFS: 2.8 months (PD-1/PD-L1 naïve urothelial and RCC), 5.2 months (PD-1/PD-L1 resistant patients), and 3.5 months (prostate cancer patients)	No DLTs reported	Used with pembrolizumab
		KA2507	NCT03008018	I	Advanced solid malignancies	*N* = 20 ORR 0%, DCR 35% with 7 SD	No DLTs reported	Used as monotherapy
	HSP90	Onalespib	NCT02503709	I	Advanced solid malignancies	*N* = 21 ORR 5%, DCR 48% with 1 PR that lasted > 10 months, 9 SD	2 DLTs: grade 3 troponin elevation and mucositis	Used with a cyclin-dependent kinase inhibitor (AT7519M)
	WEE-1	Adavosertib	NCT02617277	I	Advanced solid malignancies	*N* = 54 ORR 4%, DCR 36% with 2 PR and 17 SD	3 DLTs: nausea (*n* = 2) and diarrhea (*n* = 1)	Used with durvalumab

*AE* Adverse Event, *BC* Breast Cancer, *CK* Creatine Kinase, *CR* Complete Response, *CRC* Colorectal Cancer, *CRS* Cytokine Release Syndrome, *DCR* Disease Control Rate, *DFP* Disease-Free Progression, *DLT* Drug-Limiting Toxicities, *GGT* Gamma Glutamyl Transferase, *HCC* Hepatocellular Carcinoma, *HNSCC* Head and Neck Squamous Cell Carcinoma, *IDO* Indoleamine 2,3-dioxygenase, *MSS* Microsatellite Stable, *OS* Overall Survival, *MDS* Myelodysplastic Syndrome, *NHL* Non-Hodgkin’s Lymphoma, *NSCLC* Non-Small Cell Lung Carcinoma, *OR* Objective Response, *ORR* Objective Response Rate, *PD* Progressive Disease, *PFS* Progression-Free Survival, *PR* Partial Response, *RCC* Renal Cell Carcinoma, *RFS* Recurrence Free Survival, *SCLC* Small-Cell Lung Cancer, *SD* Stable Disease, *TLR* Toll-Like Receptor, *TNBC* Triple Negative Breast Cancer

## Stimulatory pathways

Under homeostatic conditions, stimulatory immune checkpoints promote immune activation and facilitate anti-tumor response [2]. Cancer cells block these pathways in favor of an immunosuppressive microenvironment that allows for their survival. Activation of these pathways can re-establish immune recognition of cancer cells and unleash immune anti-tumor response [9]. In this review, we will describe molecules that target T cell and other co-stimulatory pathways.

### T cell co-stimulatory targets

#### CD27 and CD70

Two signals are required for T cell activation: a T cell receptor stimulatory signal by MHC and a co-stimulatory signal [10]. Costimulatory receptors are divided into two categories, the immunoglobulin and tumor necrosis factor (TNF) receptor superfamilies [11]. CD27, a member of the TNF superfamily, is one of the most important co-stimulatory receptors. After binding its ligand, CD70 (expressed by activated dendritic, B, T, and natural killer cells), CD27 promotes T cell activation and formation of effector and memory T cells [12]. Overexpression of CD70 by tumor cells leads to chronic activation of T cells and immune exhaustion [13].

Stimulation of this pathway through activation of CD27 or blockade of CD70, enhances the efficacy of existing immunotherapies without increasing toxicity [12]. Agents targeting CD27 and CD70 are less efficacious when used alone or in poorly immunogenic microenvironments (“cold tumors”). These agents should be combined with existing immunotherapies [11].

Varlilumab is a human monoclonal antibody (mAb) that binds and enhances CD27. CD27 is located on T cells within the tumor milieu. A phase I clinical trial evaluated the use of varlilumab as monotherapy in 25 patients with advanced solid tumors (NCT01460134). Results from the dose-escalation phase of the trial, revealed an overall response rate (ORR) of 4% and a disease control rate (DCR) of 36% [14]. One partial response (PR) was seen in a patient with metastatic renal cell carcinoma (RCC). Eight patients had stable disease (SD) that lasted more than 3 months [14]. Overall, this therapy was well-tolerated. One grade 3 adverse event (AE), transient hyponatremia, was reported. The other AEs were grade 1 or 2 [14]. The trial was completed. It appears that CD27 therapy is well tolerated but additional studies are needed to determine clinical efficacy. Further evaluation of the patient with RCC may provide clues to tumor characteristics associated with response to therapy.

Varlilumab has also been used in conjunction with nivolumab in a phase I/II clinical trial in patients with advanced, treatment refractory solid malignancies (NCT02335918). Data for 90 patients, 49 with ovarian cancer and 41 with colorectal cancer (CRC), was published in an abstract [15]. The results demonstrated an ORR of 8% and a DCR of 37%. Seven PRs were seen. Two of these patients had CRC and 5 patients had ovarian cancer. Twenty-six patients had SD, 7 of which had CRC and 19 with ovarian cancer [15]. Serious AEs including acute kidney injury, mixed motor neuropathy, pneumonitis, small bowel obstruction, and acute hepatitis were observed in 3 CRC and 2 ovarian cancer patients [15]. This trial was completed and final publication is pending. While some clinical response was seen and therapy was well tolerated, it is hard to assess the role varlilumab played. Only anti-PD-1/PD-L1 naïve patients were included in the trial. PD-L1 and CD8 + T cell expression in the TME correlated with response to therapy. While on treatment, upregulation of these markers was seen more frequently among patients with ovarian cancer [15]. This may explain why responses were improved in the ovarian cancer group. Perhaps these biomarkers can be used to assess response to therapy.

ARGX-110 is an IgG1 mAb that targets CD70 on tumor and Treg cells to prevent immune exhaustion. CD70 is unable to bind to CD27 to activate T cells [16]. ARGX-110 is being used as monotherapy in a phase I/II clinical trial in patients with CD70 positive solid and hematologic malignancies (NCT01813539). Results from the dose-escalation phase of the trial were published [13]. There were 26 patients included. The DCR was 54%. There were 14 patients (12 with solid tumors and 2 with hematologic malignancies) who achieved SD with a mean duration of 3.7 months [13]. There were no dose-limiting toxicities (DLTs) reported. There were 3 grade 5 (fatal) AEs including respiratory failure, sepsis, and general health deterioration. Thirteen patients developed grade 3 or 4 toxicities including pneumonia and hemolytic anemia. The most common AEs were grade 1–2 and included fatigue and mild infusion related reactions [13]. This trial was completed. Although the authors did not attribute the grade 5 toxicities to ARGX-110, results from subsequent phases of the trial will help clarify the safety of this drug. Future research should evaluate the role of CD70 and/or CD27 as biomarkers for response to therapy.

#### CD40 and CD40L

CD40 is another member of the TNF receptor superfamily and is expressed by antigen presenting cells (APCs) including B lymphocytes, monocytes, and dendritic cells (DCs) [17]. Its ligand, on the other hand, is expressed by T helper cells. The interaction of CD40 and CD40L results in upregulation of intercellular adhesion molecules and pro-inflammatory cytokines. This leads to T cell and macrophage activation and proliferation [18, 19].

Monoclonal antibodies that enhance CD40 antitumor activity in the TME can overcome immune-checkpoint inhibitor (ICI) resistance or improve the efficacy of ICIs [19]. These therapies may increase toxicity. Cytokine release syndrome (CRS), thromboembolic events (due to the expression of CD40 by endothelial cells and platelets), thrombocytopenia, and autoimmune reactions have been reported [17, 19]. Additionally, the use of a single agent targeting CD40 is not as effective as combination therapy, however, it is unclear which combination therapy is most effective. Monotherapy may only be an option in highly mutated tumors like melanoma [19, 20]. Future research is needed to identify biomarkers that will predict response to these agents and identify those at risk for the development of toxicity [17, 19].

SEA-CD40 is a mAb that targets and stimulates CD40 on APCs. This results in T cell activation and anti-tumor effects. SEA-CD40 is being studied as monotherapy in a phase I clinical trial in patients with relapsed and/or refractory solid malignancies (NCT02376699). Results for 34 evaluable out of 48 included patients were presented in an abstract [21]. The ORR was 3% and the DCR was 32%. One patient achieved a PR and 10 had SD [21]. There were 5 DLTs, all infusion-related reactions. No grade 5 AEs were reported, however, there were several serious AEs including chills (65%), nausea (52%), dyspnea (27%), and headache (27%) [21]. This trial is open for enrollment. Further research is needed to assess the safety of this therapy. SEA-CD40 monotherapy appears to be efficacious in heavily pre-treated patients with solid malignancies and is being evaluated in hematologic malignancies [21].

CP-870,893 is a fully human mAb that stimulates CD40 on APCs. This leads to T cell activation, cytokine release, and anti-tumor response. CP-870,893 is being used in conjunction with tremelimumab, an anti-CTLA-4 agent, in a phase I clinical trial in patients with metastatic melanoma (NCT01103635). Results from 22 evaluable patients revealed an ORR of 27% and a DCR of 59%. Two patients achieved complete response (CR), 4 had PR, and 7 had SD [22]. The median progression-free survival (PFS) was 3.2 months with a median follow-up of 45 months. Nine patients survived more than 3 years. In the dose-escalation phase, 2 patients had DLTs including immune colitis. Other toxicities included grade 3 CRS (*n* = 1), grade 3 hypopituitarism (*n* = 1), grade 3 hypophysitis (*n* = 1), and grade 2 hypothyroidism (*n* = 2) [22]. Combination therapy with tremelimumab and CP-870,893 demonstrated encouraging clinical efficacy. It is unclear if the AEs were associated with CP-870,893, tremelimumab, or were a result of combination therapy. Future research could evaluate CP-870,893 in other solid or hematologic malignancies and in combination with other ICIs like anti-PD-1/PD-L1 agents.

JNJ-64457107 (JNJ-107) is an IgG1 human mAb that stimulates CD40 on APCs. It is being investigated as monotherapy in a phase I clinical trial in patients with advanced solid malignancies (NCT02829099). Preliminary results published in an abstract were available for 95 patients with a median age of 59 years [23]. The ORR was 1% and the DCR was 25%. One patient achieved a PR and 23 patients had SD. In the dose-escalation phase, there were 2 DLTs including 1 grade 3 headache and 1 grade 3 elevation of transaminases coupled with a grade 2 hyperbilirubinemia. There was 1 grade 3 and 48 grade 1 and 2 infusion-related reactions including pruritus, flushing, and rash. Other frequent AEs included fever (41%), headache (26%), and nausea (22%). This trial is open but not actively recruiting. The therapy appears to be safe and well-tolerated. Additional research combining this agent with existing ICIs or chemotherapy will help clarify its role as mono and adjunct therapy [23].

APX005M, another mAb that targets and stimulates CD40 on APCs, is being studied in combination with nivolumab in a phase I/II clinical trial in patients with melanoma and immunotherapy-naïve non-small cell lung cancer (NSCLC) (NCT03123783). Preliminary results from 19 patients were published in an abstract [24]. In the phase I portion, 9 patients were evaluated. The ORR was 22% with a DCR of 67%. Two patients had PR, 4 had SD, and 3 had progressive disease (PD). In the phase II portion, 10 additional patients were included. In these newly-recruited patients, the ORR was 20% and the DCR was 40% (2 PR, 2 SD, and 6 PD) [24]. There were no grade 4 or 5 AEs. A total of 5 grade 3 AEs were reported and included elevated transaminases, elevated bilirubin, anemia, and pneumonitis [24]. The trial was completed and final results are pending. Preliminary data is encouraging, and the drug appears to be well tolerated. This therapy increases the number of T cells and inflammatory cytokines in the TME [24]. Perhaps it could be used in patients with “cold TMEs” in an attempt to improve response to immunotherapy.

Another phase I/II clinical trial using APX005M with pembrolizumab in patients with metastatic melanoma is underway (NCT02706353). No preliminary results were available at the time of data cutoff. The trial is open and recruiting.

#### 4-1BB and 4-1BBL

4-1BB (CD137) is another member of the TNF receptor family and it is expressed by T cells, natural killer (NK) cells, B cells, monocytes, and neutrophils [25]. After binding to its ligand (4-1BBL), 4-1BB promotes activation and proliferation of these cells, including cytotoxic CD8 + T cells. This leads to enhanced direct anti-tumor response [25, 26]. Activation of NK cells and APCs also favors antibody-dependent cell-mediated cytotoxicity [26]. 4-1BB/4-1BBL therapy is promising given its pleotropic effect on both direct cytotoxicity and antibody-dependent cellular toxicity. Use of this therapy may be limited due to the broad expression of 4-1BB by non-malignant cells, increasing the risk for on-target, off-tumor toxicities (e.g., hepatitis) [27, 28].

Utomilumab (PF-05082566) is a human mAb that stimulates 4-1BB on T and NK cells, resulting in pro-inflammatory and anti-tumor activity. It has been used in conjunction with pembrolizumab in a phase I clinical trial in patients with advanced solid tumors (NCT02179918). Results available for 23 patients demonstrated an ORR of 26% and a DCR of 70% [29]. Two patients achieved a CR, 4 had a PR, and 10 patients had SD. The median duration of response was not reached. In the dose-escalation phase, there were no DLTs or grade 5 toxicities. There was 1 case of grade 3 adrenal insufficiency and 1 case of grade 3 hypokalemia. The most common AEs were grade 1 and 2 and included constitutional symptoms (35%), pruritus (22%), and fever (13%) [29]. The trial was completed. The data suggests that this therapy is both efficacious and safe. It is unclear whether the response was due to the 4-1BB therapy, pembrolizumab, or combination therapy. Improved responses were seen in patients with higher CD8 + T cells in peripheral blood. Perhaps CD8 + T cell levels can be investigated to assess response to therapy.

ADG106, a fully human IgG4 mAb that stimulates 4-1BB on T and NK cells, is being studied as monotherapy in a phase I clinical trial in patients with advanced solid malignancies and relapsed/refractory non-Hodgkin’s lymphoma (NHL) (NCT03802955). Preliminary results for 15 patients (14 evaluable for response) were presented in an abstract [30]. The results revealed an ORR of 0% and a DCR of 57%. Eight patients achieved SD and 3 patients had reduction in the size of their tumor [30]. In the dose-escalation phase, there were no DLTs or grade ≥ 3 AEs reported. There was 1 serious AE (anemia) that was not attributed to ADG106. Seven patients (47%) had an AE. The AEs included rash, pruritus, fever, nausea, vomiting, and chest discomfort [30]. This trial is open and actively recruiting. Finalized results will determine the benefit of this agent as monotherapy. Future research is needed to assess which malignancies respond best to this therapy and to determine if the addition of other immunotherapies results in improved outcomes.

#### OX40 and OX40L

OX40 (CD134) is another member of the TNF receptor family that is expressed by activated CD4+, CD8+, Treg cells, and to a lesser degree by neutrophils and NK cells. The OX40 ligand (OX40L) is expressed on activated APCs, NK cells, and mast cells [31]. Upon interaction, the OX40/OX40L pathway leads to enhanced activation, proliferation, survival, and cytokine production of CD4 + T, CD8 + T, and NK cells [31, 32]. OX40 has been found on the surface of tumor infiltrating lymphocytes (TILs) in different malignancies, including head and neck squamous cell carcinoma (HNSCC), CRC, gastric, breast and ovarian cancers. Increased OX40 expression correlates with improved outcomes [32, 33]. Targeting this pathway enhances humoral and cytotoxic antitumor responses through activation of CD4+, CD8+, and NK cells [32]. Unfortunately, the presence of myeloid-derived suppressor cells (MDSCs) within the TME impairs CD40 activity. This may explain its limited efficacy when used as monotherapy [31, 33]. At this time, there are no markers to determine which patients will benefit from these drugs.

MEDI0562 is a humanized mAb that targets and stimulates OX40 on T and NK cells. It was used in a phase I clinical trial with either durvalumab or tremelimumab in patients with advanced, refractory solid malignancies (NCT02705482). Results of 58 patients (27 treated with durvalumab and 31 with tremelimumab) were recently published in an abstract [34]. Among the 26 evaluable patients in the durvalumab cohort, the ORR was 12% and the DCR was 46%. Three patients achieved a PR and 9 had SD. Among the 31 patients in the tremelimumab cohort, the ORR was 0% and the DCR was 29%. Nine patients had SD. In the dose-escalation phase, there were 5 cases of DLTs (2 with durvalumab and 3 with tremelimumab) but no specifics were provided. There were 2 grade 5 AEs related to MEDI0562 including renal failure (durvalumab cohort) and multiorgan failure (tremelimumab cohort). In addition, grade 3 and 4 AEs were reported in both cohorts (74% in durvalumab and 68% in tremelimumab). Twelve patients discontinued MEDI0562 due to these AEs. The most common toxicities included fatigue (56%) and pruritus (45%) [34]. The trial was completed and final publication is pending. Preliminary data showed promising results, particularly in the MEDI0562 and anti-PD-L1 combination cohort. Toxicity remains a concern. Future research can determine if this therapy is safe and can also assess if combination of OX40 agents with ant-PD-L1 therapy is superior to anti-CTLA-4 agents.

GSK3174998 (GSK998), a humanized IgG1 mAb that targets and stimulates OX40 on T cells, is being investigated as monotherapy or in combination with pembrolizumab in a phase I clinical trial in patients with previously treated, advanced solid malignancies (NCT02528357). Results for 138 patients including 45 on monotherapy and 96 on combination therapy were published in an abstract [35]. The ORR was 7% and the DCR was 14%. Two patients achieved a CR in the combination cohort. Eight patients achieved a PR (1 monotherapy and 7 combination) and 10 achieved SD (1 monotherapy and 9 combination) [35]. In the dose-escalation phase, there were 2 DLTs reported in the combination group including 1 grade 3 pleural effusion and 1 grade 1 myocarditis. The other AEs were grade 1 and 2 and included nausea, diarrhea, and fatigue [35]. This trial was completed, and final publication is pending. Therapy appeared to be well tolerated and clinical response was seen. Results of subsequent phases will determine if OX40 agents are effective as monotherapy. In addition, it will help assess if this therapy can augment response in immunotherapy-refractory disease as suggested by the preliminary data [35]. OX40 levels are associated with response to therapy. Selecting patients with elevated OX40 expression may result in better clinical outcomes and should be explored further [35].

ATOR-1015 is a bi-specific mAb with the ability to bind both CTLA-4 and OX-40. This agent inhibits the immunosuppressive effects of CTLA-4, often overexpressed by T cells within the TME. In addition, ATOR-1015 promotes OX40-mediated T cell activation which leads to inflammation and anti-tumor activity [36]. It is being evaluated as monotherapy in a phase I clinical trial in patients with advanced solid malignancies (NCT03782467). Preliminary results for 15 patients with a median age of 52 years and a median of 6 lines of therapy were presented in 2 abstracts [37, 38]. Although no specific data regarding response is provided, the authors mention that 6 patients remain enrolled. Nine patients discontinued therapy as a result of confirmed PD (*n* = 1), clinical deterioration (*n* = 6), or death from PD (*n* = 2). There were no DLTs reported in the dose-escalation phase, and no grade 3–5 toxicities were seen. There were 6 patients who developed grade 1 and 2 AEs (no specifics provided). Four patients developed infusion-related reactions including abdominal pain, rash, and vitiligo [37, 38]. The trial is active but not recruiting. Final results will help assess safety of this therapy. In addition, it will evaluate the utility of combining OX40 with anti-CTLA-4 agents in heavily pretreated disease.

mRNA-2416 is a lipid nanoparticle that contains messenger RNA (mRNA) that encodes for OX40L. Intratumoral delivery of mRNA leads to translation and expression of OX40L by the tumor cell. OX40L binds OX40 on T cells which results in immune activation and tumor cell death within the TME. A phase I clinical trial is investigating mRNA-2416 as monotherapy or in combination with durvalumab in patients with advanced solid or hematologic malignancies (NCT03323398). Preliminary results were published in an abstract [39]. Results from 39 patients in the monotherapy cohort revealed an ORR of 0% and a DCR of 15% [39]. Six patients achieved SD that lasted at least 14 weeks [39]. Four patients had shrinkage of their injected tumors. In the dose-escalation portion, there were no DLTs reported. Six patients developed grade 3 AEs, however, no specifics were provided [39]. This trial is open and actively enrolling patients. The results will assess the role of monotherapy versus combination therapy with OX40L. It would be interesting to compare the efficacy of OX40 to OX40L agents. Future trials could evaluate combination of these two therapies to further enhance response.

#### GITR and GITRL

Glucocorticoid-induced TNF receptor family-related protein (GITR, [CD357]) is a receptor expressed by Tregs and activated effector T cells. Its ligand, GITRL, is expressed by APCs including DCs, macrophages, and B cells [40]. The interaction of GITR with its ligand leads to T cell activation, differentiation, and proliferation. It also inhibits Treg suppressive function [40]. Both Tregs and activated T cells play an important role in the TME. Manipulation of this pathway may alter the Treg-to-effector-T cell ratio and favor immune antitumor effects [40, 41]. Although this could help overcome immunotherapy resistance, the use of these agents relies on an abundance of Tregs and effector-T cells in the TME. Given the heterogeneity of TMEs among cancers, responses may vary with different tumors [40]. In addition, as with other immunotherapies, combination strategies may be more effective but result in increased toxicities [40].

MK-1248, a humanized mAb that stimulates GITR on T cells, is being investigated in a phase I clinical trial. It is being used as monotherapy or in combination with pembrolizumab in patients with advanced solid tumors (NCT02553499). Thirty-seven patients with CRC, melanoma, and RCC were studied. Twenty patients received MK-1248 alone and 17 received combination therapy. In the monotherapy cohort, the ORR was 0% and the DCR was 15%. There were no CR or PR [42]. Three patients had SD. In the combination cohort, the ORR was 18% and the DCR was 47%. There was 1 patient with CR, 2 with PR and 3 patients with SD [42, 43]. No DLTs were identified in the dose-escalation phase of the trial. The majority of patients (36/37 or 97%) developed at least 1 AE. Nineteen patients (51%) experienced grade 3–5 toxicities including anaphylactoid reactions and mucositis [42]. All other AEs were low-grade and included gastrointestinal toxicities, fatigue, and fever [43]. The study was terminated due to program prioritization and was not a result of safety concerns. The use of MK-1248 therapy appears to have limited efficacy when used alone but perhaps improved efficacy when combined with other agents. It is overall well tolerated. The authors did notice an increased prevalence of lymphocytes within the TME among responders [42]. Future efforts could identify biomarkers that help select patients more likely to benefit from this therapy.

AMG 228 is another humanized mAb that binds to and stimulates GITR on T cells. It was studied as monotherapy in 30 patients with advanced solid tumors in a phase I clinical trial (NCT02437916). Among the 27 evaluable patients, the ORR was 0% and the DCR was 23%. Seven patients had SD. The remainder had PD [44]. According to the authors, the dose expansion phase was not initiated given the low activity seen [44]. In the dose-escalation phase, there were no DLTs. There was 1 grade 5 pneumonitis attributed to the drug. Most AEs were grade 1 and 2 (60%) and included fatigue (13%), infusion-related reaction (7%), and hypophosphatemia (7%) [44]. The trial was terminated due to business reasons and low activity of the drug as mentioned above. These results suggest that combination strategies are more likely to result in clinical response.

BMS-986156 is a monoclonal antibody that stimulates GITR on T cells. It is being investigated as monotherapy and in combination with nivolumab in a phase I/II clinical trial in patients with advanced solid malignancies (NCT02598960). Data for 292 patients was published [45]. The patients were divided into two groups: monotherapy (*n* = 34; 16 women and 18 men; median age 56.6 years) and combination therapy (*n* = 258; 140 women and 118 men; median age 60 years) [45]. In the monotherapy group, the ORR was 0% and the DCR was 32%. Eleven patients had SD. The remaining patients had either PD (*n* = 18) or non-evaluable disease (*n* = 5). In the combination group, the ORR was 7% and the DCR was 41%. Two patients achieved a CR, 19 had a PR, and 84 had SD. The rest of the patients had either PD (*n *= 117) or non-evaluable disease (*n* = 18) [45]. There was 1 DLT reported in the dose-escalation phase of the trial. This was a grade 4 elevation in creatine kinase. There were no grade 5 AEs. There were no grade 3–4 AEs reported in the monotherapy group. Twenty-four grade 3–4 AEs (9%) were reported in the combination group. These included colitis, infusion reactions, and pancreatitis. The most common AEs were grade 1 and 2. There was 1 serious grade 2 pneumonitis reported in the monotherapy group [45]. The trial was recently completed. The data suggested that use of combination therapy with anti-PD-1 agents resulted in improved response, however, more grade 3–4 toxicities were seen. Further research is needed to assess the differences among responders and non-responders. As suggested previously, perhaps cancers with lymphocyte-rich TMEs are more likely to benefit. In addition, future efforts could evaluate this agent in hematologic malignancies.

### Other direct immune stimulatory targets

#### LXR

Liver X receptors (LXRs) are nuclear receptors involved in cholesterol synthesis and transportation, glucose balance, and fatty acid metabolism [46]. LXRs also inactivate and deplete MDSCs [47]. LXR stimulation causes increased activation and proliferation of T cells, enhanced recognition of malignant cells by macrophages, and decreased tumor angiogenesis, growth, and metastasis [47–49]. Although LXRs are an attractive target for immune modulation, their effects on cholesterol and glucose metabolism raise concerns about their safety and cardiovascular toxicities [50].

RGX-104 is an oral molecule that targets and stimulates LXR-β on the surface of immune cells. This promotes the transcription of apolipoprotein E which leads to MDSC inactivation, activation of cytotoxic T cells, and systemic pro-inflammatory and anti-tumor immune response [51]. RGX-104 is being studied as monotherapy or in combination with docetaxel in a phase I clinical trial in patients with advanced solid or hematologic malignancies (NCT02922764). Preliminary results were published in an abstract [52]. Among 12 patients who received monotherapy, the DCR was 42%. There were 5 confirmed cases of SD after 8 weeks of treatment and 1 unconfirmed PR [52]. One patient with a neuroendocrine tumor had a 53% reduction in the metastatic load [52]. There was evidence of cytotoxic lymphocyte activation and DC stimulation in 10 patients. Three patients developed grade 3 or 4 neutropenia and 1 patient had an episode of grade 3 hypercholesterolemia that improved with statin therapy. No other AEs were reported [52]. This trial is open for enrollment. It is too early to assess efficacy of this therapy; however, 1 patient did achieve a significant reduction in their tumor burden. Perhaps additional research can investigate unique patient and/or tumor characteristics which could account for this response.

The preliminary results from the combination cohort of this same trial were presented separately in another abstract [51]. Of those included, 9/11 patients were response evaluable. The ORR was 22% and the DCR was 66%. Two patients achieved CR, both with ICI refractory disease. Four patients had SD and 1 of these had a durable response lasting more than 14 weeks [51]. In the dose-escalation portion of the trial, neutropenia was reported as a DLT, but the number of affected patients was not included. Details regarding other AEs were not disclosed, however, the authors mentioned these were consistent with the individual toxicity profiles of docetaxel and RGX-104 [51]. It appears response rates did improve with combination therapy. Additional research could evaluate the best combination strategy. Perhaps RGX-104 could be used to overcome resistance to immunotherapy in ICI-refractory disease.

#### IL-2

IL-2 is a cytokine that induces T cell proliferation, differentiation, and activation against tumor cells [53]. It is secreted by activated T cells and acts in an autocrine and paracrine fashion [54]. IL-2 exerts its effects through IL-2 receptor (IL-2R) which is composed of three chains: α (expressed on Tregs), β and γ (expressed by effector T, NK, and memory CD8 + T cells) [55, 56]. IL-2 is considered the first immunotherapy effective against human cancer and was approved by the FDA for metastatic RCC in 1992 and metastatic melanoma in 1998 [56]. In spite of its anticancer activity, there is concern that it may also be immunosuppressive given its dual role as an activator of effector T cells and Tregs [55, 56]. In addition, IL-2 is rapidly metabolized, has a short half-life, and has been associated with severe toxicities which limit its use. These include vascular leak syndrome, pulmonary edema, and cardiotoxicity [56].

RO6874281 is a fusion protein consisting of a mAb bound with engineered IL-2. The antibody component is targeted against fibroblast activation protein (FAP), a molecule overexpressed by fibroblasts within the TME [57]. The IL-2 portion selectively binds IL-2Rβγ and activates CD8 + T and NK cells within the TME [58]. This agent is being studied as monotherapy in a phase I clinical trial in patients with metastatic solid tumors (NCT02627274). Preliminary results for 35 patients were presented in an abstract [58]. Long-lasting (> 6 months) responses were seen in 3 patients (1 HNSCC, 1 penile squamous cell carcinoma, and 1 melanoma) [58]. The details of these responses were not provided. There was evidence of activation and expansion of NK and effector T cells but not of Tregs in patients treated with RO6874281. Most AEs were grade 1 and 2 and included constitutional symptoms, infusion related reactions, and transaminitis [58]. The trial is open and recruiting. Early results suggest this therapy is well tolerated. Additional studies are needed to confirm the effects of RO6874281 on Tregs. Supportive data could alleviate concerns regarding IL-2 immunosuppressive effects.

hu14.18-IL2 is another fusion protein consisting of a mAb linked to IL-2. The antibody component targets GD2, a molecule overexpressed in several solid malignancies. The IL-2 component binds IL-2R and activates T and NK cells within the TME [59]. hu14.18-IL2 was studied as monotherapy in a phase II clinical trial in patients with resectable stage III and stage IV melanoma (NCT00590824). Results were available for 18 patients [60]. Of the 18 patients, 11 received hu14.18-IL2 neoadjuvant therapy and 7 received adjuvant therapy. No specific details regarding response were provided. The median recurrence-free survival (RFS) was 5.7 months, and the median OS was 61.6 months [60]. Grade 3–4 AEs leading to dose-adjustment included hypotension (*n* = 3), syncope (*n* = 1), elevated aspartate aminotransferase (*n* = 2), and elevated serum creatinine (*n* = 1) [60]. Other common AEs were grade 1 and 2 and included hypotension, transient cytopenias, hyperglycemia, and hypophosphatemia [60]. This trial was completed and results show prolonged clinical response to IL-2 therapy [60]. Interestingly, higher levels of TILs in the TME correlated with longer response and a trend towards improved overall survival. Further research should assess the use TILs as a biomarker for response to IL-2 therapy [60].

ALKS 4230 is an engineered fusion protein composed of a circularly permuted IL-2 and IL-Rα. It selectively binds IL-2Rβγ on CD8 + T and NK cells resulting in the activation of these cells and their anti-tumor activity [61]. ALKS 4230 is being evaluated as monotherapy and in combination with pembrolizumab in a phase I/II clinical trial in patients with advanced solid malignancies (NCT02799095). Preliminary results for 56 patients, including 36 receiving monotherapy and 20 receiving combination therapy were published in 2 abstracts [61, 62]. In the monotherapy cohort, 14 patients were evaluable for response. The ORR was 0% and the DCR was 57%. Eight patients had SD with 1 extending beyond 6 months. In the combination cohort, 11 patients were evaluable for response. The ORR was 9% and the DCR was 73%. One patient achieved PR and 7 had SD [62]. There were no reported DLTs in the dose-escalation portion of the trial. Eleven patients developed grade 3–4 AEs, most notably transient leukopenia. All other AEs were grade 1–2 and included fever (75%) and chills (72%) [62]. This trial is open and actively recruiting. Preliminary results are encouraging from a safety standpoint which may be due to the specificity of the engineered IL-2 for IL-2Rβγ. Further research is needed to assess its safety when used alone and in combination with other ICIs. It would also be important to assess whether this selectivity affects clinical response.

Bempegaldesleukin (NKTR-214–BEMPEG) is an engineered, pegylated IL-2 that selectively binds and activates IL-2Rβγ on CD8 + T and NK cells. CD8 + T and NK cell activation promotes antitumor response within the TME. Bempegaldesleukin is being investigated in combination with nivolumab in a phase I/II clinical trial in patients with advanced solid malignancies (NCT02983045). Preliminary results published in an abstract were available for 34 patients of which 23 were evaluable for response. The ORR was 48% and the DCR was 70%. Four patients achieved CR, 7 had PR, and 5 had SD [63]. Therapy was discontinued in 9% of patients due to AEs. There were no grade 4–5 AEs. Grade 3 AEs were seen in 18% of patients. The most common AEs were grade 1 and 2 and included fatigue (59%), fever (38%), chills (32%), and flu-like symptoms (32%) [63].

A subset analysis of 41 previously untreated stage IV melanoma patients, 38 of which were evaluable, was published in an abstract [64]. These patients received combination therapy with bempegaldesleukin and nivolumab [64]. The ORR was 53%. Thirteen patients achieved CR and 7 had PR. There was no mention of SD. The median time to response was 2 months with a median time to CR of 7 months. The median duration of response was not reached [64]. There were 4 patients (10%) who discontinued therapy due to AEs. Six patients experienced grade ≥ 3 AEs, although no details were provided [64]. This trial is active but not recruiting. Preliminary results suggest that combination therapy with nivolumab is well tolerated. Response to therapy was seen regardless of PD-L1 tumor expression [63, 64]. Perhaps this therapy can be used to enhance response to ICIs in cancers with low-PD-L1 expression.

IRX-2 is a human-derived, cell-free mixture of cytokines including IL-2, IL-1 and IL-8. These cytokines bind with their receptors to activate immune cells within the TME. IRX-2 is being studied in combination with nivolumab in a phase I clinical trial in patients with advanced, refractory solid malignancies (NCT03758781). No preliminary results are available. This trial is active and enrolling patients.

#### IL-15

IL-15 is a potent molecule produced by DCs, macrophages, and monocytes [65]. After binding to its receptor (IL-15R), IL-15 promotes activation and proliferation of effector T cells, B cells, and NK cells [65]. It has a limited effect on Tregs which is thought to be an advantage over IL-2 therapy. Unfortunately, IL-15 can exacerbate pre-existing autoimmune disease including rheumatoid arthritis, sarcoidosis, nephritis, and inflammatory bowel disease [66, 67]. In addition, its antiapoptotic effect has been associated with the growth of leukemic cells. In particular, progression of human T cell lymphotropic virus (HTLV-1)-associated adult T cell leukemia/lymphoma, acute lymphocytic lymphoma, and chronic lymphocytic leukemia have been reported [65–67]. Elevated serum IL-15 levels have been associated with Alzheimer’s disease [66]. In addition to these potential AEs, the short half-life and poor bioavailability may limit its use [66].

ALT-803 is a fusion protein composed of an IgG bound to a mutated IL-15. The mutated IL-15 has increased biologic activity and acts as a superagonist of IL-15R. This leads to activation of NK and effector T cells with pro-inflammatory and anti-cancer effects [68]. ALT-803 is being used in combination with nivolumab in a phase I clinical trial in patients with stage III or IV NSCLC (NCT02523469). Results for 21 patients revealed an ORR of 29% and a DCR of 76%. Six patients achieved a PR and 10 had SD [68]. The median PFS was 9.4 months, and the median OS was 17.4 months. Of the 21 patients, 10 had PD and 8/10 died as a result of their disease. In the dose-escalation portion of the trial, there were no DLTs or grade 4–5 toxicities reported. Grade 3 AEs were noted in 2 patients. One patient developed lymphopenia and the other had a myocardial infarction. The remaining AEs were grade 1–2 and included injection site reactions (19/21 or 90% of patients) and flu-like symptoms (15/21 or 71% of patients) [68]. The trial is active but no longer recruiting. These results suggest that IL-15 may be safe and efficacious when used with nivolumab. Response to therapy was seen in ICI-refractory NSCLC [68]. Further studies should assess for similar response in other malignancies.

Another phase I/II clinical trial evaluated the use of ALT-803 as monotherapy in patients with relapsed hematologic malignancies after receiving allogenic bone marrow transplantation (NCT01885897). Thirty-three patients were evaluated. The ORR was 6% and the DCR was 15%. One patient achieved a CR that lasted 7 months. One patient had a PR lasting 5 months. There were 3 cases of SD lasting at least 2 months [69]. There were no DLTs in the dose-escalation portion of the trial. Two patients developed grade 4 sepsis secondary to neutropenia from active leukemia. One patient experienced a fatal intracranial bleed from disease-related thrombocytopenia. None of these severe or fatal AEs were attributed to the medication. The most common AEs were grade 1, 2, and 3. They included local skin reactions and constitutional symptoms [69]. This trial was completed. The results suggest IL-15 therapy may be beneficial and safe for use in hematologic malignancy. In the future, ALT-803 could be evaluated in combination with other therapies in an attempt to enhance response. In addition, it would be important to assess if prior bone marrow transplantation reduced response to this therapy.

n-803 is another IL-15 superagonist consisting of a mutated IL-15 with increased biologic activity bound to an IgG. It activates NK and effector T cells to promote anticancer activity [70]. n-803 is being investigated in a phase I clinical trial as monotherapy in healthy volunteers (NCT03381586). Results were available for 14 individuals. n-803 produced a remarkable increase in NK cells that persisted for at least 24 days. In addition, there were increased levels of inflammatory cytokines including interferon gamma, IL-10, and IL-6 [71]. There were no grade 3, 4, or 5 toxicities reported. The most common AEs included grade 1 and 2 injection site reactions, fever, and chills [71]. The trial was completed. The therapy appears to be well tolerated in this small study. The changes seen in the TME could be further investigated in cancer patients.

A recombinant human IL-15 (rhIL-15) is being studied in conjunction with nivolumab and ipilimumab in a phase I clinical trial in patients with advanced solid malignancies (NCT03388632). No preliminary results were available. The trial is open and actively recruiting.

#### A3R

Adenosine is a molecule with multiple physiologic effects. It serves as a backbone of ATP. It also helps maintain tissue homeostasis, controls inflammation, and promotes healing [72]. Under physiologic conditions, extracellular levels of adenosine are low. Adenosine levels increase dramatically in response to tissue injury and help modulate inflammatory response. This response varies depending on the adenosine receptor stimulated [72]. Receptors like A2aR and A2bR primarily mediate immunosuppressive responses, while A1R and A3R lead to immune activation [73].

A3R is expressed by both non-immune (e.g. brain, lung, testes) and immune cells (e.g. eosinophils, APCs, lymphocytes) [74]. Inflammatory conditions are associated with overexpression of A3R on activated T cells [74, 75]. Elevated levels of A3R have been found in multiple malignancies including CRC and hepatocellular carcinoma (HCC). Activation of A3R is thought to affect cancer cell proliferation and is the main death-inducing receptor found on tumor cells [74, 76]. A3R agonists serve as potential immunotherapy agents. It is unclear who might benefit from this therapy and if overexpression of A3R correlates with response [74].

Namodenoson (CF102) is an oral selective molecule that directly binds and stimulates A3R on the surface of cancer cells inducing apoptosis [77]. It is being investigated as monotherapy in a phase II clinical trial in patients with advanced, refractory Child–Pugh class B HCC (NCT02128958). Results for 78 patients were available in an abstract [78]. Fifty patients were treated with namodenoson and 28 received placebo [78]. Among the placebo group, the ORR was 0%. Among the 34 evaluable patients in the namodenoson cohort, the ORR was 9%. Three patients achieved an objective response. No additional information regarding the type or duration of response was provided. The OS was 4.3 months for patients treated with placebo. The OS was 4.1 months for patients receiving namodenoson. In a subgroup analysis, the median OS was 6.8 months with a PFS of 3.5 months in those with Child–Pugh score of 7 treated with namodenoson. Those with a Child–Pugh score of 7 treated with placebo had an OS of 4.3 months and a PFS of 1.9 months [78]. Grade 3 toxicities included anemia, hyponatremia, and fatigue. Most AEs were low-grade and included nausea, peripheral edema, and elevated aspartate aminotransferase (AST) [78]. The trial is ongoing but not recruiting. Response appears to be limited to a small subset of patients but is well tolerated. Additional research is needed to assess clinical benefit and patient characteristics associated with response. The use of namodenoson in other malignancies could also be explored.

#### CD11b/CD18

CD11b interacts with CD18 to form a multifunctional surface receptor known as CD11b/CD18 or Mac-1. It is expressed by multiple immune cells including neutrophils, monocytes, macrophages, DCs, and NK cells [79]. Binding of a ligand to CD11b, the ligand-binding subunit of the receptor, results in enhanced cellular adhesion, migration, chemotaxis, phagocytosis, and cytotoxicity [79]. Several malignancies, including pancreatic adenocarcinoma, have large numbers of dysfunctional myeloid cells within the TME [80]. Agents stimulating CD11b have resulted in improved anti-tumor myeloid cell function, enhanced T cell activity, and improved response to chemotherapy, radiation, and immunotherapy [81]. CD11b activation is a promising strategy to overcome ICI therapy resistance [81]. In theory, TMEs with abundant myeloid-cells or tumor-associated macrophages (TAMs) are needed for this therapy to work. It is uncertain if tumors with less TAMs will derive benefit.

GB1275 is a first-in-class oral modulator of CD11b. It reduces MDSCs and increases active TAMs and CD8 + T cells within the TME [82]. GB1275 is being investigated in a phase I/II clinical trial in advanced, refractory solid malignancies. It is being used as either mono- or combination therapy with pembrolizumab or nab-paclitaxel plus gemcitabine (NCT04060342). Preliminary results from 22 patients (14 receiving monotherapy and 8 receiving combination therapy with pembrolizumab) were recently presented in an abstract [82]. At time of cutoff, the DCR was 32% with 7 patients achieving SD (4 from the monotherapy cohort and 3 from the combination cohort). A reduction of circulating MDSCs was observed in the majority of patients [82]. There were no DLTs in the dose-expansion portion of the trial. There were no severe AEs reported. Three patients discontinued therapy due to death from their underlying disease [82]. Nine patients developed grade 1 AEs. These included dysesthesia, constipation, nausea, decreased appetite, photosensitivity, and fatigue [82]. The trial is open and enrolling patients. This therapy appears to be well-tolerated. Further research to assess the efficacy of GB1275 as standalone and adjunct therapy is needed.

#### STING

Stimulator of interferon genes (STING) is a protein with signaling properties located on the membrane of the endoplasmic reticulum (ER) of both non-immune and immune cells [83, 84]. DNA from the mitochondria and nucleus of cells is released in response to bacterial and viral infections or death of healthy and malignant cells. The presence of DNA in the cytoplasm is recognized as a danger signal. The DNA is detected by a DNA-sensing protein, cyclic GMP–AMP synthase (cGAS), leading to the production of cyclic GMP-AMP (cGAMP). cGAMP binds STING resulting in a conformational change that leads to its activation [84, 85]. Upon activation, STING travels to the nucleus where it promotes transcription and production of pro-inflammatory cytokines and type I interferon [84]. These cytokines lead to maturation and activation of DCs and T cells. In addition, STING induces direct activation of T cells, B cells and NK cells and has antineoplastic properties [83, 84]. It promotes cytotoxic and humoral responses and enhances immune cell trafficking and effector T cell infiltration of the TME. It also augments tumor antigen presentation and directly triggers cancer cell death [83, 84]. STING is often underexpressed in cancer cells (e.g. CRC, melanoma, ovarian cancer) and has become an attractive target in immune-oncology [83]. Constitutive activation of STING has been associated with autoimmune disorders and raises concern regarding the safety of this therapy [84]. In addition, growing evidence suggests that STING activation may have immunosuppressive effects. This can be due to blockade of T cell activation, induction of T cell death, activation of immunosuppressive cells (e.g. MDSCs, Tregs), and upregulation of negative immune regulators such as IL-10, IDO, PD-1, and PD-L1 [84, 86].

MIW815 (ADU-S100) is a synthetic cyclic dinucleotide that stimulates STING to promote the release of pro-inflammatory and anti-tumor cytokines [87, 88]. It is being studied as intratumoral therapy in combination with spartalizumab, an anti-PD-1 agent, in a phase I clinical trial in patients with advanced solid malignancies and lymphomas (NCT03172936). Preliminary results for 66 patients with a median age of 61 years were published in an abstract [88]. PR was seen in immunotherapy-naïve triple-negative breast cancer (TNBC) and immunotherapy-resistant melanoma patients, but no specifics were provided [88]. At the time of cut off, 74% of patients (*n* = 49) had been unenrolled from the trial either because of PD (*n* = 28), physician decision (*n* = 18), AEs (*n* = 2), or death (*n* = 1). In the dose-escalation phase, no DLTs were reported. Four patients developed grade 3–4 elevation of transaminases. Serious AEs included fever (2%), increased amylase/lipase (4%), diarrhea (2%), partial seizures (2%), and pneumonitis (2%). Other common but mild AEs included injection site pain (12%), fever (12%), and diarrhea (9%) [88]. This trial is active but not recruiting. Finalized results will help assess the safety and efficacy of this therapy. These results may also assess response in patients with ICI-refractory disease, particularly in the melanoma population. Evaluation of the TME and circulating cytokines among responders could also help identify potential biomarkers for response.

SB 11,285, a stimulator of STING, is being studied alone and in combination with nivolumab in a phase I clinical trial in patients with advanced solid malignancies (NCT04096638). No preliminary results are available. The trial is open and actively recruiting.

#### TLR

Toll-like receptors (TLRs) are important mediators of immune activation against infection, non-infectious inflammation, and tissue repair. The role of TLRs in cancer is complex [89]. Stimulation of TLR-1, 2, and 6 has been associated with activation of effector-T cells, release of pro-inflammatory cytokines, and suppression of Tregs. TLR-2 is also expressed by cancer cells and has been associated with increased vascularization, tumor invasion, and progression of disease [90]. TLR-3 mediates T cell activation and pro-inflammatory cytokine release. Additionally, it has direct anti-tumor properties and promotes cancer cell death. TLR-4 has been associated with the activation of APCs, B cells and T cells [91]. TLR-4 has also been linked with pro-tumor effects through promotion of angiogenesis and tumor invasion [90]. TLR-5 enhances the activity of DCs and effector T cells, and promotes the secretion of pro-inflammatory cytokines. It also induces tumor cell apoptosis [90]. TLR-7, TLR-8 and TLR-9 activate effector T cells and induce pro-inflammatory cytokines (e.g. IL-2 and IL-10) that promote antitumor effects [90]. In addition, TLR-9 sensitizes tumors to radiation [90]. In order to utilize TLR therapies, special attention is required to ensure anticancer pathways are being stimulated and protumor pathways are being inhibited [92]. In addition, the use of TLR therapies may be limited in highly immunosuppressive TMEs [93].

Tomaralimab (OPN-305) is a fully humanized mAb that inhibits TLR-2 expressed by malignant cells. It was studied as monotherapy in a phase I/II clinical trial in heavily pretreated patients with low/intermediate risk myelodysplastic syndrome (MDS) (NCT02363491). Results were available in an abstract [94]. There were 22 evaluable patients out of 51. The median age was 72 years and the patients were predominantly male (79%) [94]. The ORR was 50% and the DCR was 73%. There were 6 patients who achieved CR (transfusion independence), 5 with PR, and 5 with SD. There were no DLTs reported in the dose-escalation phase of the trial. No additional toxicity data was provided [94]. This trial was completed. A large percentage of patients appeared to respond to therapy. Further studies should assess use of this drug in hematologic and solid malignancies.

G100 is a glucopyranosyl lipid A that stimulates TLR-4 and promotes activation of APCs and T cells. A phase I clinical trial used intratumoral G100 monotherapy in patients with unresectable or metastatic soft tissue sarcomas (NCT02180698). Preliminary results for 14 evaluable patients (out of 15 included) with superficial lesions were published in an abstract [95]. The ORR was 14% and the DCR was 100%. There was 1 patient with CR, 1 with PR, and 11 with SD. Among the 3 patients with long-term follow-up, the mean duration of response was 235 days. There were no grade 3–5 AEs reported, however, no additional safety data was provided [95]. The trial was completed. The authors noted an on-treatment increase of CD4 + T cells within the TME. In addition, they mention that pre-treatment TNF-α corresponded with PFS [95]. Perhaps TNF-α can be explored as a biomarker for response to therapy in future studies.

NJH395 is an immune-stimulator antibody conjugate consisting of a TLR-7 agonist bound to an anti-HER2 antibody. It enhances pro-inflammatory/anti-tumor responses against HER2-expressing malignant cells while limiting systemic toxicities [96]. A recent first-in-human phase I clinical trial evaluated NJH395 monotherapy in patients with non-breast HER2 + advanced malignancies (NCT03696771). Preliminary results from the study were published in an abstract [96]. Eighteen patients were included (10 males and 8 females) with a median age of 52 years. The majority of patients had CRC (*n* = 11). The ORR was 0% and the DCR was 50%. No patients achieved CR or PR. There were 9 patients with SD. In the dose-escalation portion of the trial, there were 5 DLTs reported including 3 cases of increased liver enzymes, 1 aseptic meningitis, and 1 meningism. The incidence of AEs was 94% and the most common grade 3–4 AEs included lymphopenia (28%) and increased liver enzymes (11%). Other common AEs reported included CRS, fever, nausea/vomiting, and headache [96]. The trial was completed. The treatment appears to be toxic and did not demonstrate significant clinical benefit. Further studies could assess the use of this therapy in HER2 + breast cancer [96].

Motolimod (VTX-2337), a potent TLR-8 agonist, is being studied in conjunction with cetuximab in a phase I clinical trial in patients with previously untreated stage II, III, and IV HNSCC (NCT02124850). Results for 14 patients demonstrated an enhanced inflammatory response within the TME. Increased active monocytes, decreased Treg function, and reduced immunosuppressive markers (e.g., CTLA-4 and CD73) were seen [97]. No efficacy data was reported. There were no grade 4 or 5 toxicities. Most AEs were grade 1 and 2 and included acneiform dermatitis (79%), injection site reactions (79%), and flu-like symptoms (36%) [97]. This trial was terminated for unclear reasons.

A phase II clinical trial evaluated motolimod in combination with platinum therapy, fluorouracil, and cetuximab in patients with recurrent or metastatic HNSCC (NCT01836029). Results for 195 patients with a median age of 58 years were published [98]. One hundred patients were randomized to receive chemotherapy with motolimod and 95 received chemotherapy with placebo. Among the patients who received motolimod, the ORR was 40% and the DCR was 62%. There were 2 patients with CR, 36 with PR, and 22 had SD. Fifty-six patients (56%) had documented PD. Fifty-four patients died of their disease. The median PFS was 6.1 months, and the median OS was 13.5 months. Among the placebo cohort, the ORR was 34% and the DCR was 58%. There were 5 patients with CR, 27 with PR, and 23 with SD. The PFS was 5.9 months and the OS was 11.3 months [98]. The authors concluded that the addition of motolimod did not add any statistically significant improvement to the ORR, PFS, or OS [98]. In a subgroup analysis of human papilloma virus (HPV) positive patients, however, motolimod improved PFS (7.8 months) and OS (15.2 months) when compared to placebo (5.9 and 12.6 months respectively) [98]. In addition, patients with injection site reactions demonstrated improved PFS and OS. Serious AEs from the motolimod cohort included vomiting (6%), pneumonia (6%), and dehydration (6%). Irrespective of grade, injection site reactions (39%), chills (37%), fever (43%), dermatitis acneiforme (48%), and anemia (60%) were more commonly reported in the motolimod cohort. The incidence of all other AEs was comparable among both groups [98]. This trial was completed. Although results failed to demonstrate substantial clinical benefit of motolimod, the results of the HPV positive cancers were intriguing. Perhaps future research can evaluate motolimod with ICIs in this subset of patients.

MEDI9197 is a lipophilic molecule that binds to and stimulates TLR-7 and 8. It was administered intratumorally to minimize systemic toxicities. MEDI9197 promotes recruitment and activation of cytotoxic T and NK cells within the TME [99]. A first-in-human phase I clinical trial evaluated intratumoral use of MEDI9197 with or without durvalumab and/or radiation therapy in patients with advanced solid malignancies (NCT02556463). Results were recently published [100]. There were 52 patients enrolled: 35 received MEDI9197 monotherapy and 17 patients received MEDI9197 with durvalumab. There were 5 patients in the monotherapy cohort, and 2 in the combination cohort who also received radiation. The ORR was 0% in both cohorts, and the DCR was 29% in the monotherapy and 18% in the combination cohort. No CRs or PRs were seen, however, 10 patients had SD in the monotherapy group and 3 had SD in the combination group [100]. In the dose-escalation phase, there were 2 DLTs in the monotherapy cohort consisting of CRS. There was 1 DLT in the combination cohort due to hemorrhagic shock from a ruptured liver metastasis. Across both cohorts, leukopenia accounted for the majority of the grade 3–4 AEs. Other common, non-severe AEs included fatigue, fever, and nausea [100]. The clinical trial was terminated as a result of a change in company strategy. Clinical response was limited. In spite of the intratumoral administration of this therapy, there was evidence of systemic immune activation in the form of CRS. Assessment of the TME for TLR-7/8 expression could be further explored to determine if there is a relationship between TLR levels and response to therapy.

NKTR-262 is a small molecule and agonist of TLR-7/8. It is administered intratumorally to enhance pro-inflammatory cytokine release and to recruit and activate T/NK cells. A phase I clinical trial used NKTR-262 in conjunction with an IL-2 agonist, bempegaldesleukin, in patients with relapsed/refractory advanced and metastatic solid malignancies (NCT03435640). Preliminary data was recently published in an abstract [101]. There were 36 patients enrolled but efficacy data was only available for 17 patients with melanoma. The ORR was not provided, but the DCR was 41%. No data was available regarding the number of patients and specific responses. The authors mention there were 2 PRs in patients with PD after receiving 2 previous lines of immunotherapy [101]. In the dose-escalation phase of the trial, there was 1 DLT consisting of transaminitis. The most common AEs included flu-like symptoms, fatigue, nausea, and pruritus and were attributed to bempegaldesleukin. No additional toxicity data was reported [101]. The trial remains active but is no longer recruiting. Based on the preliminary data, combination therapy was safe and effective. The authors observed an increase in circulating CD4+, CD8+, and NK cells in patients treated with NKTR-262 plus bempegaldesleukin. If these changes are seen in the TME, perhaps this approach can be used to promote a “hot” TME and enhance response to ICIs. The trial is expanding to assess the efficacy of NKTR-262 plus bempegaldesleukin with nivolumab in patients with relapsed/refractory metastatic melanoma [101].

Tilsotolimod (IMO-2125) is a synthetic TLR-9 agonist. It is being evaluated in 2 clinical trials (1 phase I and 1 phase I/II) as intratumoral monotherapy in patients with advanced metastatic solid tumors (NCT03052205, NCT02644967). Results for 51 evaluable out of 54 patients were published in an abstract [102]. The ORR was 0% and the DCR was 29%. Fifteen patients had SD. One of these patients had a clinical reduction in tumor size by 35%, however, this was not confirmed with scans [102]. There were no DLTs reported in the dose-escalation phase of the trial. The most common AEs included fatigue, fever, chills, injection site reactions, nausea, and vomiting [102, 103]. Both trials were completed, and final results are pending publication. The therapy appeared to be well tolerated. The authors mention that use of this therapy results in an increase of inflammatory markers within the TME, including immune checkpoint upregulation [102]. Additional research is needed to confirm these findings. Tilsotolimod could be used to improve response to existing ICIs.

Lefitolimod (MGN1703) is another synthetic DNA-based TLR-9 agonist. It targets TLR-9-positive DCs and potentiates immune-mediated tumor death. It was evaluated as maintenance monotherapy in a phase II clinical trial in patients with extensive-stage small-cell lung cancer (SCLC) (NCT02200081). Of the 103 patients enrolled, 62 received lefitolimod [104]. There were 59 response evaluable patients. The ORR was 12% and the DCR was 51%. While no patients achieved CR, 7 patients had PR and 23 had SD. There were no grade 5 AEs, but 5 patients developed grade 4 neutropenia. There were only 11 cases of grade 3 AEs including headache, neutropenia, and cough. The remaining AEs were grade 1–2 and included cough, headache, fatigue, and rash [104]. This trial was completed. The therapy was well tolerated, and clinical response was seen. In particular, improved responses were seen in patients with chronic obstructive pulmonary disease and those with reduced levels of active CD86 + B cells [104]. Further research could explore the role of CD86 + B cells as a biomarker for response to therapy.

Lefitolimod is being evaluated with ipilimumab in a phase I clinical trial in patients with advanced solid malignancies (NCT02668770). No results were available. The trial is active but not recruiting.

Cavrotolimod (AST-008) is a spherical nucleic acid-based TLR-9 agonist that enhances the activity of effector T and NK cells to promote antitumor effects. It is being studied as an intratumoral therapy in conjunction with pembrolizumab or cemiplimab, anti-PD-1 agents, in a phase I/II clinical trial in patients with advanced solid malignancies (NCT03684785). No results were available. This trial is actively recruiting. Finalized results will help assess safety and efficacy of combination therapy with TLR-9 agents.

#### SMAC and IAP

Inhibitor of apoptosis (IAP) proteins, including X-linked IAP (XIAP) and cellular IAP (cIAP) 1 and 2, are molecules that bind and inactivate caspase proteins and prevent apoptosis. Second mitochondrial activator of caspase (SMAC) is an endogenous protein that is found in the mitochondria. When activated and released, it enables apoptosis by binding and blocking the activity of IAPs [105]. IAPs inhibit activation and proliferation of B cells, T cells, and NK cells [106]. These proteins are overexpressed in cancer. They are associated with tumor progression, treatment failure, and poor prognosis [107].

SMAC agonists can be used to inhibit IAP and promote cancer cell death. In addition, SMAC agonists can augment response to other forms of therapy (e.g. radiation, chemotherapy, immunotherapy) by enhancing immune activation [105, 106, 108]. At this time, there are no biomarkers to help determine which patients will benefit from this therapy [106]. Further evaluation to identify the ideal target and combination therapy is needed [106].

Birinapant is a peptidomimetic of SMAC and inhibitor of IAPs that promotes apoptosis in cancer cells. It is being studied in combination with pembrolizumab in a phase I/II clinical trial in patients with advanced solid malignancies (NCT02587962). Preliminary results for 18 evaluable out of 19 patients were published in an abstract [109]. The ORR was 11% and the DCR was 22%. Two patients achieved PR and 2 had SD [109]. In the dose-escalation phase of the trial, there was 1 DLT due to grade 3 elevation in transaminases. There were no grade 4 or 5 AEs. There were 2 serious AEs consisting of grade 2 elevation of lipase. Other common, non-severe AEs were rash (*n* = 3) and stomatitis (*n* = 1) [109]. This trial was recently terminated due to futility. Preliminary analysis showed the medication was safe. Moving forward, SMAC agonists could be investigated with other ICIs and therapies.

### Indirect immune activators

In addition to direct manipulation of stimulatory immune checkpoints, there is interest in pathways that indirectly activate the immune system. The agents included here induce tumor cell death and increase antigen expression. This enhances immune recognition of cancer cells and may augment the effects of immune checkpoint therapy.

#### CXCL12 and CXCR4

CXCL12, also known as stromal cell-derived factor-1 (SDF-1), is a potent chemokine produced by stromal cells including fibroblasts and endothelial cells. After binding to its receptor, CXCR4, it activates various pathways that enhance angiogenesis, migration, proliferation, and survival [110]. CXCR4 is constitutively expressed by a wide variety of normal tissues including lymphocytes, brain, and spleen. Elevated expression has been seen in breast, lung, and prostate cancers [110]. CXCL12 is elevated in the TME and is produced by tumor-associated fibroblasts [111]. Cancer cells use the CXCL12/CXCR4 axis to promote their growth, survival, invasion, and metastasis [111, 112].

CXCL12/CXCR4 blockade directly targets malignant cells and can augment the effects of existing ICI therapy [111, 112]. Blockade of this path could impair normal wound healing and tissue repair, particularly in myocardial infarction [113]. It may also affect the interaction with other chemokines (e.g. CXCR3, CXCL11, CXCL10, CXCL9). The consequences of this disruption are unknown [114].

NOX-A12 is a pegylated mirror-image RNA oligonucleotide that targets and neutralizes CXCL12 within the TME. This interaction induces apoptosis within cancer cells and increases the activity of other anticancer therapies. It is being studied as monotherapy and in combination with pembrolizumab in a phase I/II clinical trial in patients with metastatic pancreatic and microsatellite-stable CRC (NCT03168139). Results for 20 heavily pretreated patients (9 pancreatic and 11 CRC) were published in 2 abstracts [115, 116]. The ORR was 0% and the DCR was 25%. Five patients had SD [115, 116]. The median PFS was 1.87 months. The OS was 42% at 6 months and 22% at 12 months. Although no specifics were provided about toxicity, the authors mentioned the AEs were related to pembrolizumab or the underlying disease and were not due to NOX-A12 [115, 116]. The trial was completed. Recent data revealed NOX-A12 therapy was associated with upregulation of effector T cells within the TME. Interestingly, this also appeared to correlate with SD [117]. Additional studies are required to verify these findings.

Balixafortide is an oral agent that is a highly selective inhibitor of CXCR4. Blockade of the CXCL12/CXCR4 axis within the TME, promotes tumor cell apoptosis and enhances other anticancer therapies. Balixafortide was studied in conjunction with eribulin in a phase I clinical trial in patients with metastatic, HER-2 negative, CXCR4 positive breast cancer (NCT01837095). Results were presented in a manuscript and an abstract [118–120]. Data was available for 56 heavily pretreated patients of which 54 were response evaluable [118, 119]. The ORR was 30% with a median duration of 3.2 months. The clinical benefit rate (defined as DCR with SD > 6 months) was 44% with a median duration of 6.9 months [118, 119]. The DCR was 76%. There were 16 patients who achieved a PR and 25 had SD, 8 of which lasted more than 6 months. The median PFS was 4.6 months, and the median OS was 16.8 months. Serious AEs were reported in 38% of patients. These included febrile neutropenia (9%), urinary tract infection (5%), and pneumonia (4%). There were 2 patients who died during the study, 1 from septic shock and 1 from pneumonia. These were not considered grade 5 AEs [119]. There were 15 cases of grade 4 AEs which included neutropenia, lymphopenia, and febrile neutropenia. The other AEs were grades 1–3 and commonly included fatigue (79%), infusion-related reactions (48%), constipation (46%), alopecia (46%), nausea (45%), neutropenia (34%), and anemia (29%) [119, 120]. The trial was finalized. Overall, the therapy was well tolerated, and clinical benefit was seen. The authors mention that combination therapy may be superior to eribulin monotherapy [118]. Future research is needed to confirm these findings.

#### PI3K

Phosphoinositide 3-kinase (PI3K) pathways play an important role in cell survival. Specifically, class I PI3K protein kinases (PI3Kα, PI3Kβ, PI3Kδ, and PI3Kγ) control cell growth, proliferation, and apoptosis. PI3K is upregulated in many cancer cells including colon, breast, and ovarian [121]. PI3K inhibitors have been developed to target cancer cells and the TME. PI3K blockade improves vessel function and enhances both drug delivery and migration of immune cells. This therapy may enhance response to existing immunotherapies [121].

PI3K inhibitors have been ineffective when used alone. This is due to high rates of resistance among cancer cells and the cytostatic nature of the therapy [121]. As a result, PI3K agents should be used as adjuncts. PI3Kα is critical for glucose homeostasis. Use of this therapy may be limited due to the risk of hyperglycemia and hyperinsulinemia [121].

IPI-549 is an oral selective inhibitor of PI3Kγ. It targets TAMs to induce a switch from an immunosuppressive to an immune-activating phenotype with anti-tumor effects [122]. IPI-549 is being investigated in conjunction with nivolumab in a phase I clinical trial in patients with advanced solid tumors (NCT02637531). Results published in an abstract were available for 30 patients and revealed an ORR of 7% [122]. There were 2 PRs (1 adrenocortical and 1 gallbladder carcinoma) at 8 weeks [122]. In the dose-escalation phase, there were no treatment-related deaths, but there were 2 DLTs: 1 grade 3 rash and 1 grade 3 transaminitis. Most of the AEs were grade 1 and 2 and included rash (23%), nausea, asymptomatic transaminitis, and constitutional symptoms (6% each) [122]. This trial is active but not recruiting. Preliminary results suggest this therapy is safe. Peripheral blood samples from patients treated with IPI-549 plus nivolumab revealed elevated levels of PD-L1 and CXCL9/10 [122]. Future efforts should determine if these markers correlate with response.

SAR260301 is a selective inhibitor of PI3Kβ and has been effective in cancers with PTEN deficiency. Loss of PTEN, a tumor suppressor protein, leads to PI3Kβ upregulation [123]. SAR260301 is being evaluated as monotherapy in a phase I clinical trial in patients with advanced solid tumors (NCT01673737). Preliminary results for 21 patients (19 evaluable) revealed an ORR of 0% and a DCR of 26%. Five patients had SD [124]. In the dose-escalation phase, there were 2 reported cases of DLTs: 1 grade 3 pneumonitis and 1 grade 3 gamma-glutamyl transferase elevation. The remaining AEs were grade 1 and 2 and included diarrhea, nausea, and vomiting (14% each) [124]. The clinical trial was completed. Unfortunately, SAR260301 was metabolized quickly, and the drug was unable to adequately suppress its target pathway [124]. The therapy was well tolerated, but further research should focus on improving the pharmacokinetics of this medication [124].

#### SYK and FLT-3

Spleen tyrosine kinase (SYK) is a cytoplasmic non-receptor tyrosine kinase. It mediates immune responses by coupling the activity of immune receptors with downstream intracellular pathways. For example, it promotes FMS-like tyrosine kinase-3 (FLT-3) activation [125]. SYK plays a vital role in the development, differentiation, and activation of immune cells [126]. It also plays a role in oncogenesis by promoting tumor cell proliferation and survival [127, 128]. SYK overexpression has been found in gliomas [129]. Inhibition of this pathway has been used to enhance response to other immunotherapies. In practice, however, use of these agents is challenging due to the paradoxical anti-neoplastic (via immune activation) and pro-tumorigenic effects of the pathway [130]. In addition, these therapies may affect other tyrosine kinases and result in off-target toxicities [125].

TAK-659 is a dual SYK and FLT-3 inhibitor that targets SYK-expressing tumor cells. It is being studied with nivolumab in a phase I clinical trial in patients with advanced solid tumors (NCT02834247). Preliminary results from 19 patients with breast, ovarian, colon, and pancreatic cancers revealed an ORR of 5%. There was 1 PR and 11 patients had PD. No additional information was provided [131]. In the dose-escalation phase, there were 3 cases of DLTs. One included grade 3 fever and the other 2 cases were thought to be from nivolumab. They included myocarditis and left ventricular dysfunction [131]. Therapy was poorly tolerated. Grade 3 or 4 toxicities were seen in 74% of patients. These toxicities included elevated lipase and anemia (20% each), fever (*n* = 3), nausea and sepsis (*n* = 2 for each) [131]. The trial was terminated due to concerns of limited efficacy. Perhaps this is related to the paradoxical pro- and anti-tumor effects that may result from blockade of this pathway. Further research is needed to evaluate the use of this therapy in clinical practice. Future evaluation could include hematologic malignancy.

#### MNK 1/2

The eukaryotic initiation factor 4E (eIF4E) is a cap-binding subunit of the eukaryotic initiation complex 4F. eIF4E is the rate-limiting step in the initiation of mRNA translation [132]. Under normal conditions, levels of eIF4E are low. Elevated levels of eIF4E allow for increased transcription and translation of growth factors, anti-apoptotic proteins, pro-angiogenic factors, and motility proteins (c-myc, cyclin-D1, VEGF, Bcl-2, SNAIL, and β-catenin) [132, 133].

MAPK-interacting kinases (MNK) 1 and 2 phosphorylate and activate eIF4E. MNK 1 and 2 upregulation leads to eIF4E overexpression and oncogesis [132]. eIF4E is overexpressed in a variety of malignancies. It mediates tumorigenesis and tumor progression by increasing transcription of proteins that promote proliferation, angiogenesis, and prevent apoptosis [134]. eIF4E also promotes PD-L1 expression by tumor cells [135]. MNK 1 and 2 mediate cytokine production. These cytokines (e.g. IL-1β, IL-6, MCP-1, RANTES, TNF-α) regulate cancer initiation, progression, and development of chemotherapy resistance [136].

Inhibition of MNK 1 and 2 results in downregulation of PD-L1 and also augments the effects of existing immunotherapies [133, 135]. These agents have limited utility when used as monotherapy. The appropriate combination strategy has not yet been elucidated and further investigation is needed [132]. The use of this therapy may also be limited due to on-target, off-tumor toxicities and disruption of normal mRNA translation in non-malignant cells.

Tomivosertib (eFT508) is a potent oral inhibitor of MNK 1 and 2. It prevents eIF4E phosphorylation and activation and leads to downregulation of PD-L1 in the TME. It is being investigated in combination with other ICIs in a phase II clinical trial in patients with solid malignancies. These patients must have poor response after at least 12 weeks of anti-PD1/PD-L1 therapy (NCT03616834). Preliminary results from an abstract were published [137]. Thirty-nine patients with a median age of 68 years and a median of 2 previous lines of therapy were evaluated [137]. The ORR was 5% and the DCR 46%. There were 3 patients who achieved PR and 15 with SD. In addition, 7 patients with NSCLC remained free from progression for at least 24 weeks [137]. There were 7 cases of DLTs including hypersensitivity, hepatic toxicity, and constipation. It is unclear, however, which parameters were used by the authors to define a DLT event. There were 4 cases of grade 5 AEs, but none were attributed to tomivosertib. Thirty-four patients experienced an AE from tomivosertib. Grade 3–4 AEs including elevation of transaminases, elevation of creatine kinase, and rash were seen in 11 patients. The remainder of AEs were grades 1–2. The most commonly reported were gastrointestinal symptoms including nausea, vomiting and diarrhea [137]. This trial is active but not recruiting. Early results suggest combination therapy is well tolerated and may benefit patients with suboptimal response to anti-PD-1/PD-L1 agents. Additional investigation is needed to assess the efficacy of tomivosertib as adjunct therapy.

#### HDAC

Histone deacetylases (HDAC) are a group of enzymes that help maintain chromatin structure. Under normal circumstances, DNA is wrapped around histones to form chromatin fibers. In order for DNA to undergo transcription, balance between two types of enzymes is required: histone acetyltransferases (HAT) and HDAC. HDACs remove acetyl groups which increase the binding of DNA to histones. This results in a tighter chromatin structure and reduced DNA transcription [138]. HATs add the acetyl groups back leading to a looser chromatin structure and increased DNA transcription.

HDACs are divided into 4 groups. Class I HDACs are primarily located in the nucleus and include HDACs 1, 2, 3, and 8. Class II HDACs are located in the cytoplasm and nucleus. They are subdivided into Class IIa (HDACs 4, 5, 7, and 9) and Class IIb (HDACs 6 and 10) HDACs [139, 140]. HDAC6 can deacetylate tubulin and plays an important role in cytoskeleton regulation and cell migration [141]. Class III HDACS are structurally distinct and are known as sirtuins. These are located in the nucleus, cytosol, and mitochondria and include sirtuins 1–7 [142]. Class IV HDACs are found in the nucleus and cytoplasm and include HDAC11 [139, 140, 143].

In cancer, the HAT/HDAC balance is disrupted. Increased HDAC levels promote a denser chromatin structure. As a result, there is reduced transcription of anti-oncogenic genes resulting in reduced tumor suppressors, cell-cycle inhibitors, differentiation factors, and inducers of apoptosis. This favors the development of an oncogenic phenotype and progression of cancer [144]. In addition, HDACs favor epigenetic silencing of genes within immune cells in the TME. Genes coding for MHC class I/II molecules, co-stimulatory molecules (e.g., CD40, B7-1, B7-2, ICAM-1), activating cytokines (e.g., IL-10), and proteins regulating expansion, activation, and differentiation of immune cells are downregulated. These negatively impact the function of APCs, macrophages, T cells and NK cells [138]. Class I and II HDACs, also known as “classical HDACs,” are involved in oncogenesis [140]. Elevation in HDAC 1, 6, and 8 is associated with an invasive phenotype in breast cancer [145].

HDAC inhibition directly affects oncogenesis, increases neoantigen expression by cancer cells, promotes pro-apoptotic molecules, and enhances the function of immune cells [138]. Use of this therapy is limited given differences in acetylase activity among T cell subpopulations. This could lead to downregulation of pro-inflammatory cytokines in some T cells, while enhancing activation, differentiation, and survival in others [146]. The pro-inflammatory and anti-tumor effects of other immunotherapies could be negatively impacted.

HDAC inhibitors can be selective or non-selective (pan-inhibitors) [139]. HDAC inhibitors can have various toxicities depending on the selectivity of the therapy, the location of the HDAC (e.g., nucleus, cytoplasm, mitochondria), and the tumor type [147]. The use of non-selective HDAC inhibitors has resulted in severe gastrointestinal and cardiac toxicities. They have also been associated with increased PD-L1 and PD-L2 expression by tumor cells [148]. Perhaps, selective HDAC inhibitors will improve the toxicity and efficacy profiles of these agents [148].

Vorinostat is a non-selective HDAC inhibitor. It downregulates pro-tumor gene transcription and increases expression of anti-tumor genes to promote cell cycle arrest and apoptosis in cancer cells [149]. Vorinostat is being evaluated in combination with pembrolizumab in a phase I clinical trial in patients with advanced, refractory urothelial, renal, and prostate carcinomas (NCT02619253). Results for 37 evaluable out of 43 patients were published in an abstract [150]. The ORR was 5%. There were 2 patients with PR, but no cases of SD were mentioned. Overall, the PFS was 2.8 months for PD-1/PD-L1 naïve urothelial and renal cancer, 5.2 months for PD-1/PD-L1 resistant patients, and 3.5 months for prostate cancer patients [150]. In the dose-escalation phase, there were no DLTs. There were no grade 5 AEs reported. Grade 3–4 AEs were reversible and included kidney injury, anemia, diarrhea, and hypothyroidism. The most common AEs were grade 1 and 2 and included fatigue and nausea [150]. The trial is active but not recruiting. Initial results suggest therapy is well tolerated. Clinical benefit was seen in a subset of patients with PD-1/PD-L1-refractory disease [150]. Additional research is needed to confirm these results and assess differences among responders.

KA2507 is an oral HDAC6 inhibitor. It directly affects cancer cell growth and alters the TME to enhance the effects of other immunotherapies [151]. It is being studied as monotherapy in a phase I clinical trial in patients with advanced, refractory solid malignancies (NCT03008018). Preliminary results in 20 patients with a median age of 56 years were published in an abstract [151]. The ORR was 0% and the DCR was 35%. There were 7 patients with SD. Two of these patients had SD lasting more than 12 months [151]. There were no DLTs in the dose-escalation phase. AEs were reported in 17 patients (85%). Only 5 of these were attributed to KA2507. No specifics regarding the severity and type of AEs were provided [151]. The trial was completed and final publication is pending. Preliminary results suggest that selective HDAC inhibitors are well tolerated. There is evidence that this therapy may promote a “hot” TME and increase PD-L1 expression in cancer cells [148]. Further research is needed to confirm these findings and assess for improved response with combination therapy.

#### HSP90

Heat shock proteins (HSPs) 90 and 70 are important intracellular chaperones that assist with protein transportation. They also assist with folding of protein, unfolding of protein, and prevention of protein precipitation under stressful conditions [152]. HSP90 plays an important role in bridging innate and adaptive immune response. It facilitates antigen presentation. It is important for APC and lymphocyte activation and maturation [153]. Cancer cells overexpress HSPs to facilitate their survival, growth, proliferation, and metastasis [152].

Initially, HSP90 inhibition was thought to promote cancer growth given its pro-inflammatory effect. After further evaluation, it appears that blockade of HSP90 has direct effect on cancer cells and enhances immune activation against cancer [152]. HSP90 downregulation leads to increased tumor antigen expression and upregulation of HSP70. HSP70 acts as a chemokine to recruit T cells [153]. HSP90 blockade, therefore, results in enhanced T cell killing of tumor cells and potentiates immune checkpoint therapy [154]. One benefit of this therapy is the relative overexpression of HSP90 on malignant cells. This could help decrease the incidence of on-target off-tumor effects [153]. This therapy is not without its risks. Long-term HSP90 blockade can result in increased DNA mutation frequency and decreased levels of tumor protective proteins such as LATS1 and 2. In addition, some abnormal proteins like mutant retinoblastoma proteins are degraded by HSP90. Inhibition of HSP90 could increase the risk for early-onset multifocal retinoblastoma in vulnerable populations [155]. These agents are not effective when used alone but may have a role when combined with other therapies [153].

Onalespib is an oral HSP90 inhibitor. It is being investigated in conjunction with a cyclin-dependent kinase inhibitor (AT7519M) in a phase I clinical trial in patients with advanced solid malignancies (NCT02503709). Preliminary results from 21 evaluable out of 28 patients were presented in an abstract [156]. The ORR was 5% and the DCR was 48%. There was 1 PR that lasted more than 10 months. Nine patients had SD [156]. It is unclear what medication was responsible for which AE. In the dose-escalation phase, there were 2 DLTs reported as grade 3 troponin elevation and mucositis. There were no grade 4 or 5 AEs reported. The other grade 3 toxicities included diarrhea, anemia and neutropenia. The most common AEs were grade 1–2 and included diarrhea, mucositis, nausea, vomiting, and fatigue [156]. The trial is active but not recruiting. Preliminary results were encouraging; however, it is difficult to assess which drug contributed to the response. Two patients (1 CRC and 1 endometrial cancer) continued on the study drug for more than 10 cycles with SD [156]. Additional research is needed to assess the tumor characteristics of those who benefited from HSP90 therapy.

#### WEE-1

WEE-1 is a tyrosine kinase that serves as a G2-M cell cycle checkpoint. It prevents the initiation of mitosis in the presence of DNA damage. This allows the cell to repair genomic damage that may have escaped detection at prior checkpoints [157]. G1 checkpoint dysregulation is common among cancer cells and allows for accumulation of mutations. WEE-1, however, prevents tumor cells from accumulating excessive DNA damage that would otherwise trigger apoptosis [157]. In addition, WEE-1 prevents cancer cell death when exposed to granzyme B released by cytotoxic/anti-tumor T and NK cells [158, 159]. It also phosphorylates and activates HSP90 [155].

WEE-1 blockade could lead to tumor cell death. The initiation of mitosis would trigger apoptosis from an excessive accumulation of mutations within the cancer cell. WEE-1 inhibition would also improve response to other immunotherapies. Cancer cells would be susceptible to T/NK cell-mediated death. Increased tumor antigen expression from cell death would allow for better immune recognition [157, 159]. Finally, WEE-1 inhibitors could enhance anti-HSP90 and anti-HDAC therapies [155, 160]. HDACs maintain chromatin structure upstream of WEE-1 [160].

While this therapy sounds promising, there are limitations to its use. For one, it is unclear what on-target, off-tumor effects will result in healthy tissue and progenitor cells [157]. WEE-1 blockade could increase the mutational burden within malignant cells and make them more resistant to therapy. In addition, the cells could divide more readily with inhibition of yet another cell cycle regulator [157]. It is unclear if these agents can cross the blood brain barrier for use in central nervous system disease [157].

Adavosertib is an oral tyrosine kinase inhibitor of WEE-1. It impairs the G2 DNA damage checkpoint and promotes apoptosis in heavily-mutated cancer cells. It is being investigated in conjunction with the anti-PD-L1 agent durvalumab in a phase I clinical trial in patients with advanced solid malignancies (NCT02617277). Preliminary results were published in an abstract [161]. These were available for 54 patients with colon, lung, and breast cancer. The ORR was ~ 4% and the DCR was 36%. Two patients had PR and 17 had SD. In the dose-escalation phase, there were 3 DLTs reported including nausea (*n* = 2) and diarrhea (*n* = 1). There were 7 serious AEs reported. Two of these cases consisted of reversible liver injury. Thirty-four patients (63%) experienced grade ≥ 3 AEs. Fatigue (15%), diarrhea (11%), and nausea (9%) were the most common [161]. The trial is active but not recruiting. Early data is suggestive of antitumor effect and therapy is tolerable with no drug-drug interaction [161]. It is difficult to determine the added benefit of adavosertib to durvalumab. Future research could assess the use of WEE-1 therapy alone and in combination with other agents, e.g., anti-HSP90 or anti-HDAC therapies.

## Conclusion

In contrast to traditional chemotherapy and radiation, immunotherapy utilizes the host immune system to target cancer cells. As a result, these therapies are better tolerated and, in some cases, allow for long-lasting response. The management of cancer has changed with the development of these agents. Only a small percentage of patients respond to the currently approved immune therapies. Efforts are focused at improving the efficacy and application of these drugs. As we continue to advance our understanding of the immune system, we can better manipulate these pathways to enhance their anti-cancer effects. This can be achieved by directly activating T effector and other immune cells, altering the immunosuppressive TME, or targeting DNA to enhance antigen expression on cancer cells.

While new therapies targeting these pathways have shown promising results, they too have limitations in their clinical application. In general, many of these therapies have been used to augment existing immune therapies and are not efficacious when used as monotherapy. The synergistic effects of these agents can increase toxicities and immune-related AEs. In addition, on-target off-tumor toxicities, CRS, metabolic dysregulation, and secondary malignancies have been reported. There are no biomarkers to predict clinical response or development of side effects. In the future, research efforts focused on development of these biomarkers will allow for a more tailored approach to treatment and will help elucidate the best combination therapies. The future of immune therapy is bright and will continue to improve outcomes in cancer patients.

## Data Availability

All data generated or analyzed during this study are included in this published article.

## Abbreviations

AE: Adverse event
APCs: Antigen-presenting cells
AST: Aspartate aminotransferase
CR: Complete response
CRC: Colorectal cancer
CRR: Complete response rate
CRS: Cytokine release syndrome
CTLA-4: Cytotoxic T lymphocyte-associated molecule-4
DCR: Disease control rate
DCs: Dendritic cells
DLTs: Dose-limiting toxicities
eIF4E: Eukaryotic initiation factor 4E
ER: Endoplasmic reticulum
FLT-3: FMS-like tyrosine kinase-3
GITR: Glucocorticoid-induced TNF receptor family-related protein
GITR-L: GITR ligand
HAT: Histone acetyltransferases
HCC: Hepatocellular carcinoma
HDAC: Histone deacetylases
HIV: Human immunodeficiency virus
HNSCC: Head and neck squamous cell carcinoma
HPV: Human papilloma virus
HSP: Heat shock proteins
HTLV-1: Human T cell lymphotropic virus
IAP: Inhibitor of apoptosis
ICIs: Immune checkpoint inhibitors
LXRs: Liver X receptors
mAb: Monoclonal antibody
MDS: Myelodysplastic syndrome
MDSCs: Myeloid-derived suppressor cells
MHC: Major histocompatibility complex
MNK: MAPK-interacting kinases
mRNA: Messenger RNA
NHL: Non-Hodgkin's lymphoma
NKs: Natural killers
NSCLC: Non-small cell lung cancer
ORR: Objective response rate
OS: Overall survival
PD: Progressive disease
PD-1: Programmed cell death receptor-1
PD-L1: Programmed cell death receptor-1 ligand
PFS: Progression-free survival
PI3K: Phosphoinositide 3-kinase
PR: Partial response
RCC: Renal cell carcinoma
RFS: Recurrence-free survival
SCLC: Small-cell lung cancer
SD: Stable disease
SMAC: Second mitochondrial activator of caspase
STING: Stimulator of Interferon genes
SYK: Spleen tyrosine kinase
TAMs: Tumor-associated macrophages
TILs: Tumor infiltrating lymphocytes
TLR: Toll-like receptor
TME: Tumor microenvironment
TNBC: Triple-negative breast cancer
TNF: Tumor necrosis factor
Tregs: Regulatory T cells
XIAP: X-linked IAP
